# Data on synthesis and structure–activity relationships of tetrazolato-bridged dinuclear platinum(II) complexes

**DOI:** 10.1016/j.dib.2021.107697

**Published:** 2021-12-10

**Authors:** Seiji Komeda, Hiroki Yoneyama, Masako Uemura, Takahiro Tsuchiya, Miyuu Hoshiyama, Tomoya Sakazaki, Keiichi Hiramoto, Shinya Harusawa

**Affiliations:** a Faculty of Pharmaceutical Sciences, Suzuka University of Medical Science, Suzuka, Mie 513-8670, Japan; b Department of Pharmaceutical Organic Chemistry, Osaka Medical and Pharmaceutical University, Takatsuki, Osaka 569-1094, Japan

**Keywords:** Platinum, Tetrazole, Cancer, NMR, MS

## Abstract

In this data file, the synthetic procedures for the preparation of a series of anticancer tetrazolato-bridged dinuclear platinum(II) complexes ([{*cis*-Pt(NH_3_)_2_}_2_(μ-OH)(μ-5-R-tetrazolato-*N*2,*N*3)]^n+^ (*n* = 1 or 2, tetrazolato-bridged complexes)) and of the bridging ligands of 5-substituted 1*H*-tetrazoles (5-R-1*H*-tetrazoles) are described. These compounds were characterized by ^1^H-, ^13^C-, ^19^F- and ^195^Pt-NMR spectroscopy and mass spectrometry.

## Specifications Table

**Table:** 

Subject	Chemistry
Specific subject area	Inorganic, organic and medicinal chemistry
Type of data	General protocol for synthesis with structure, NMR and MS data; in supplementary data –NMR and mass spectra.
How data were acquired	For the tetrazolato-bridged dinuclear platinum(II) complexes, the ^1^H-, ^13^C-,^19^F-and ^195^Pt-NMR spectra were recorded on (^1^H 400 MHz, Agilent, Santa Clara, CA, US) or a Varian NMR System (^1^H 600 MHz, Agilent) at 293 K. All ^1^H- and ^13^C-NMR spectra were referenced to TSP [sodium 3-trimethylsilyl-propionate-2,2,3,3-d(4), *δ* = 0], ^195^Pt chemical shifts to K_2_PtCl_4_ (*δ* = −1614), and ^19^F chemical shifts to CF_3_COOH (*δ* = −76.55). MS was performed by using a micrOTOF-Q quadrupole–time-of-flight mass spectrometer (Bruker, Billerica, MA, US) in the positive ion mode. For the 5-R-1*H*-tetrazole derivatives, ^1^H- and ^13^C-NMR spectra were measured in CDCl_3_ with tetramethylsilane (TMS) as the internal standard on a Varian Mercury-300 or Agilent 400-MR-DD2 spectrometers. ^19^F-NMR spectra were recorded at 282 MHz (Varian Mercury-300) or 376 MHz (Agilent 400-MR-DD2), and the chemical shifts were measured relative to CF_3_CO_2_H as an external standard. High-resolution mass spectrometry spectra were determined by using a JMS-700(2) mass spectrometer (JEOL Ltd., Tokyo, Japan) operating in positive-ion mode. Melting points were determined using a Yanagimoto micromelting apparatus.
Data format	Raw and analyzed.
Parameters for data collection	Data were collected for characterisation purposes.
Description of data collection	Data were collected via the raw output files from the respective hardware. ^1^H and ^13^C or ^19^F NMR spectra were recorded as fid files.
Data source location	Faculty of Pharmaceutical Sciences, Suzuka University of Medical Science, Suzuka, Japan. 34.852990, 136.586422 Department of Pharmaceutical Organic Chemistry, Osaka University of Pharmaceutical Sciences, Takatsuki, Japan. 34.864006, 135.574493
Data accessibility	With the article
Related research article	S. Komeda, H. Yoneyama, M. Uemura, T. Tsuchiya, M. Hoshiyama, T. Sakazaki, K. Hiramoto, S. Harusawa Synthesis and structure–activity relationships of tetrazolato-bridged dinuclear platinum(II) complexes: A small modification at tetrazole C5 markedly influences the *in vivo* antitumor efficacy *Journal of Inorganic Biochemistry* https://doi.org/10.1016/j.jinorgbio.2018.12.009

## Value of the Data

• The data contain the synthetic procedure for preparation of the anticancer tetrazolato-bridged dinuclear platinum(II) complexes [{*cis*-Pt(NH_3_)_2_}_2_(μ-OH)(μ-5-R-tetrazolato-*N*2,*N*3)]^n+^ (*n* = 1 or 2) and their bridging ligands, 5-R-1*H*-tetrazoles.
• The data provide valuable guidance for researchers working on inorganic, organic and medicinal chemistry, and on drug-discovery research.
• The data serve as characterization of 15 original tetrazolato-bridged complexes and 5-R-1*H* tetrazoles.
• Some tetrazolato-bridged complexes may enter clinical trial as promising anticancer drug candidates.

## 1 Data Description

**Fig. 1:**
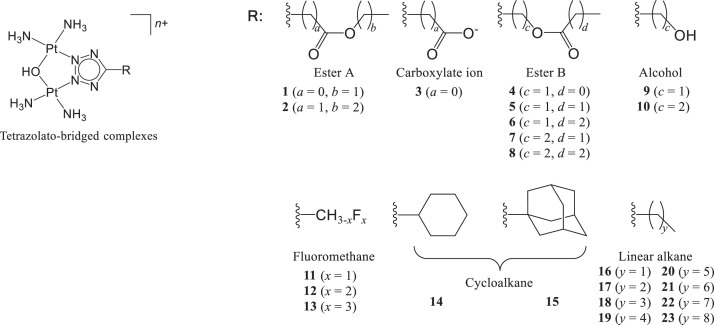
Structures of the series of tetrazolato-bridged dinuclear Pt(II) complexes, among which 15 new tetrazolato-bridged complexes were newly synthesized.

**Fig. 2:**
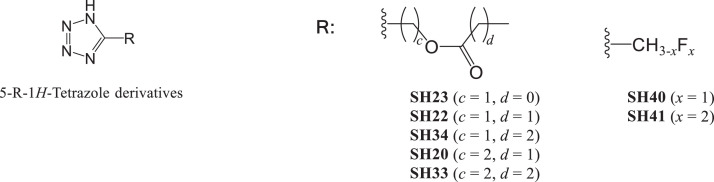
Structures of newly synthesized 5-substituted 1*H*-tetrazoles (5-R-1*H*-tetrazoles)

With a unique DNA binding mode [1], [2], [3], [4], [5], [6], [7], [8], [9] and a different cellular uptake pathway [5,10] than currently available platinum-based drugs, tetrazolato-bridged dinuclear platinum(II) complexes (tetrazolato-bridged complexes) with the general formula [{*cis*-Pt(NH_3_)_2_}_2_(μ-OH)(μ-5-R-tetrazolato-*N*2,*N*3)]^2+^ (Fig. 1) are currently being developed as next-generation platinum-based drugs, [11], [12], [13], [14] and many are reported to be effective against cancers with intrinsic [5] or acquired [3,10] resistance to platinum-based drugs. Complexes **1**–**15** were newly synthesized, along with seven 5-R-1*H*-tetrazole derivatives (**SH**; Fig. 2), which were synthesized by the reactions of sodium azide and inactive nitriles in DMF in a microwave reactor to provide efficient transformation into tetrazoles. These newly prepared compounds were characterized by using ^1^H-, ^13^C-, ^19^F- or ^195^Pt-NMR spectroscopy and mass spectrometry.

## 2 Experimental Design, Materials and Methods

### 2.1 General information

#### 2.1.1 Synthesis of tetrazolato-bridged dinuclear Pt(II) complexes

K_2_PtCl_4_ was obtained from Tanaka Kikinzoku Kogyo K.K. (Tokyo, Japan), and 5-(trifluoromethyl)-1*H*-tetrazole was purchased from Fluorochem Ltd (Hadfield, UK). The tetrazole derivatives [13] (ethyl 1*H*-tetrazole-5-carboxylate, propyl 1*H*-tetrazole-5-aceate, 5-(cyclohexyl)-1*H*-tetrazole and 5-(adamantyl)-1*H*-tetrazole) were prepared according to published methods. Other tetrazole derivatives (**SH20, SH22, SH23, SH33, SH34, SH40** and **SH41**) were newly prepared and synthetic procedures are described below. The ^1^H-NMR spectra of **1, 2, 4**-**12, 14** and **15**, ^13^C- and ^195^Pt-NMR spectra of **1**–**15**, and ^19^F-NMR spectra of **13**–**15** were recorded on (^1^H 400 MHz, Agilent, Santa Clara, CA, US) or a Varian NMR System (^1^H 600 MHz, Agilent) at 293 K and are shown in Fig. S1–S4. All ^1^H- and ^13^C-NMR spectra were referenced to TSP [sodium 3-trimethylsilyl-propionate-2,2,3,3-d(4), *δ* = 0], ^195^Pt chemical shifts to K_2_PtCl_4_ (*δ* = −1614), and ^19^F chemical shifts to CF_3_COOH (*δ* = −76.55). For **1**–**15,** MS was performed by using a micrOTOF-Q quadrupole–time-of-flight mass spectrometer (Bruker, Billerica, MA, US) in the positive ion mode, and the mass spectra are shown in Fig. S5.

#### 2.1.2 Synthesis of 5-R-1H-Tetrazoles

Microwave-assisted reactions were performed in a Milestone MultiSYNTH multimodal reactor with thermal control. Reactions with air- and moisture-sensitive compounds were carried out under an Ar atmosphere. Anhydrous solvents were purchased from WAKO Chemical Co. Solvents were removed in a rotary evaporator under reduced pressure. Fuji Silysia FL-60D silica gel was used for flash column chromatography. TLC was performed on pre-coated TLC plates (WAKO silica gel 70 F_254_). ^1^H- and ^13^C-NMR spectra were measured in CDCl_3_ with tetramethylsilane (TMS) as the internal standard on a Varian Mercury-300 or Agilent 400-MR-DD2 spectrometers. ^19^F-NMR spectra were recorded at 282 MHz (Varian Mercury-300) or 376 MHz (Agilent 400-MR-DD2), and the chemical shifts were measured relative to CF_3_CO_2_H as an external standard. High-resolution mass spectrometry spectra were determined by using a JMS-700(2) mass spectrometer (JEOL Ltd., Tokyo, Japan) operating in positive-ion mode. Melting points were determined using a Yanagimoto micromelting apparatus and were uncorrected.

### 2.2 General procedure

#### 2.2.1 Synthesis of tetrazolato-bridged dinuclear Pt(II) complexes

5-R-1*H*-tetrazole (1.79 mmol) was dissolved in 5 mL MeOH and added to a solution of [*cis*-Pt(NH_3_)_2_(µ-OH)]_2_(NO_3_)_2_ (1.0 g, 1.62 mmol) in 25 mL of water. The solution was stirred and heated at 40 °C for 24 h in the dark and then filtered; the filtrate was evaporated to dryness using a centrifugal evaporator (CVE-3000; Tokyo Rikakikai Co, Ltd). The resulting powder was collected on a glass filter, washed with 2-propanol and diethyl ether, and recrystallised from water (compound **1, 2, 4, 5**-**7, 11**) or methanol (**12, 13**) using the centrifugal evaporator. For the synthesis of **3,** a 1 M lithium hydroxide solution (300 μL) was added to a solution prepared by dissolving 0.20 g of compound **1** in 5 mL of distilled water, which was stirred for approximately 10 min at room temperature. The resulting white precipitate was filtered and washed with 2-propanol and diethyl ether, recovered by filtration, dried in a vacuum desiccator. For the synthesis of **9** or **10**, a 1 M lithium hydroxide solution (300 μL) was added to a solution prepared by dissolving 0.20 g of compound **4** or **8** in 5 mL of distilled water, and the resulting solution was stirred for approximately 10 min at room temperature. The pH of the solution was adjusted to 7 with 0.1 M aqueous nitric acid and then lyophilized. The resulting white powder was washed with 2-propanol and diethyl ether. The dried powder was re-dissolved, and the pH of the solution was adjusted to 7 with 0.1 M aqueous nitric acid, and the solution was then evaporated to dryness. This process was repeated three times to replace any remaining calboxylate ions (as counterions) with nitrate ions. The resulting white powder was washed with 2-propanol and diethyl ether. For the synthesis of **14** or **15**, a solution of 5-alkyl-1*H-*tetrazole (1.79 mmol) in 15 mL of MeOH was added to a solution of [*cis*-Pt(NH_3_)_2_(µ-OH)]_2_(NO_3_)_2_ (1.0 g, 1.62 mmol) in 30 mL of water. The solution was stirred vigorously at 50 °C for 48 h in the dark and then lyophilized. The resulting white powder was collected on a glass filter, washed with 2-propanol and diethyl ether, and recrystallized from methanol.

*[{cis-Pt(NH_3_)_2_}_2_(µ-OH)(µ-ethyltetrazolato-5-carboxylate-N2,N3)](NO_3_)_2_* (**1**)

Yield: 384 mg (32.0%). ^1^H NMR (400 MHz, D_2_O, Fig. S1.1): *δ* = 1.42 (t, 3H, *J* = 7.4 Hz), 4.52 (q, 2H, *J* = 7.2 Hz). ^13^C NMR (151 MHz, D_2_O, Fig. S2.1): *δ* = 16.1 (1C), 66.7 (1C), 159.2 (1C), 161.6 (1C). ^195^Pt NMR (129 MHz, D_2_O, Fig. S4.1): *δ* = −2186. MS (ESI, Fig. S5.1) [M-H]^+^: 615.1 (M = [{*cis-*Pt(NH_3_)_2_}_2_(µ-OH)(µ-ethyl tetrazolato-5-carboxylate*-N2,N3*)]^2+^)

*[{cis-Pt(NH_3_)_2_}_2_(µ-OH)(µ-propyltetrazolato-5-acetate-N2,N3)](NO_3_)_2_* (**2**)

Yield: 212 mg (17.0%). ^1^H NMR (400 MHz, D_2_O, Fig. S1.1): *δ* = 0.91 (t, 3H, *J* = 7.4 Hz), 1.67 (sx, 2H, *J* = 7.4 Hz), 4.12 (s, 2H), 4.16 (t, 2H, *J* = 7.4 Hz). ^13^C NMR (151 MHz, D_2_O, Fig. S2.1): *δ* = 12.4 (1C), 24.1 (1C), 33.6 (1C), 71.0 (1C), 161.8 (1C), 174.3 (1C). ^195^Pt NMR (129 MHz, D_2_O, Fig. S4.1): *δ* = −2179. MS (ESI, Fig. S5.1) [M-H]^+^: 643.1 (M = [{*cis-*Pt(NH_3_)_2_}_2_(µ-OH)(µ-propyl tetrazolato*-*5-acetate-*N2,N3*)]^2+^)

*[{cis-Pt(NH_3_)_2_}_2_(µ-OH)(µ-tetrazolato-5-carboxylate-N2,N3)](NO_3_)* (**3**)

Yield: 171 mg (89.0%). ^13^C NMR (151 MHz, D_2_O, Fig. S2.1): *δ* = 166.6 (1C), 184.9 (1C). ^195^Pt NMR (129 MHz, D_2_O, Fig. S4.1): *δ* = −2181. MS (ESI, Fig. S5.1) [M]: 587.0 (M = [{*cis-*Pt(NH_3_)_2_}_2_(µ-OH)(µ-tetrazolato-5-carboxylate*-N2,N3*)]^+^)

*[{cis-Pt(NH_3_)_2_}_2_(µ-OH)(µ-5-(acetoxy)methyltetrazolato-N2,N3](NO_3_)_2_* (**4**)

Yield: 138 mg (11.3%). ^1^H NMR (400 MHz, D_2_O, Fig. S1.2): *δ* = 2.15 (s, 3H), 5.40 (s, 2H). ^13^C NMR (151 MHz, D_2_O, Fig. S2.2): *δ* = 23.0, 59.5, 163.4, 176.2. ^195^Pt NMR (129 MHz, D_2_O, Fig. S4.2): *δ* = −2181. MS (ESI, Fig. S5.1) [M-H]^+^: 615.1 (M = [{*cis-*Pt(NH_3_)_2_}_2_(µ-OH)(µ-5-(acetoxy)methyltetrazolato-*N2,N3*]^2+^)

*[{cis-Pt(NH_3_)_2_}_2_(µ-OH)(µ-5-(propionyloxy)methyltetrazolato-N2,N3)](NO_3_)_2_ (* ***5*** *)* 

Yield: 115 mg (9.6%). ^1^H NMR (400 MHz, D_2_O, Fig. S1.2): *δ* = 1.11 (t, 3H, *J* = 7.6 Hz), 2.46 (2H, q), 5.41 (s, 2H, *J* = 7.6 Hz). ^13^C NMR (151 MHz, D_2_O, Fig. S2.2): *δ* = 11.0, 29.9, 59.4, 163.5, 179.7. ^195^Pt NMR (129 MHz, D_2_O, Fig. S4.2): *δ* = −2181. MS (ESI, Fig. S5.1) [M-H]^+^: 629.1 (M = [{*cis-*Pt(NH_3_)_2_}_2_(µ-OH)(µ-5-(propionyloxy)methyl tetrazolato-*N2,N3*)]^2+^)

*[{cis-Pt(NH_3_)_2_}_2_(µ-OH)(µ-5-(butyryloxy)methyltetrazolato-N2,N3)](NO_3_)_2_* (**6**)

Yield: 263 mg (21.5%). ^1^H NMR (600 MHz, D_2_O, Fig. S1.2): *δ* = 0.91 (t, 3H, *J* = 7.2 Hz), 1.62 (sx, 2H, *J* = 7.2 Hz), 2.43 (t, 2H, *J* = 7.2 Hz), 5.41 (2H, s). ^13^C NMR (151 MHz, D_2_O, Fig. S2.2): *δ* = 15.6, 20.7, 38.3, 59.4, 163.5, 178.9. ^195^Pt NMR (129 MHz, D_2_O, Fig. S4.2): *δ* = −2181. MS (ESI, Fig. S5.2) [M-H]^+^: 643.1 (M = [{*cis-*Pt(NH_3_)_2_}_2_(µ-OH)(µ-5-(butyryloxy)methyltetrazolato-*N2,N3*)]^2+^)

*[{cis-Pt(NH_3_)_2_}_2_(µ-OH)(µ-(propionyloxy)ethyltetrazolato-N2,N3)](NO_3_)_2_* (**7**)

Yield: 403 mg (33.0%). ^1^H NMR (400 MHz, D_2_O, Fig. S1.2): *δ* = 1.06 (t, 3H, *J* = 7.6 Hz), 2.36 (q, 2H, *J* = 7.6 Hz), 3.28 (t, 2H, *J* = 6.4 Hz), 4.50 (t, 2H, *J* = 6.4 Hz). ^13^C NMR (151 MHz, D_2_O, Fig. S2.2): *δ* = 12.6, 17.8, 35.3, 56.4, 160.5, 175.9. ^195^Pt NMR (129 MHz, D_2_O, Fig. S4.2): *δ* = −2179. MS (ESI, Fig. S5.2) [M-H]^+^: 643.1 (M = [{*cis-*Pt(NH_3_)_2_}_2_(µ-OH)(µ-(propionyloxy)ethyltetrazolato-*N2,N3*)]^2+^)

*[{cis-Pt(NH_3_)_2_}_2_(µ-OH)(µ-5-(butyryloxy)ethyltetrazolato-N2,N3)](NO_3_)_2_* (**8**)

Yield: 93 mg (6.6%). ^1^H NMR (400 MHz, D_2_O, Fig. S1.3): *δ* = 0.86 (t, 3H, *J* = 7.2 Hz), 1.55 (sx, 2H, *J* = 7.2 Hz), 2.32 (t, 2H, *J* = 7.2 Hz), 3.29 (t, 2H, *J* = 6.4 Hz), 4.51 (t, 2H, *J* = 6.4 Hz). ^13^C NMR (151 MHz, D_2_O, Fig. S2.3): *δ* = 15.6, 20.8, 27.6, 38.5, 65.2, 165.4, 179.6. ^195^Pt NMR (129 MHz, D_2_O, Fig. S4.3): *δ* = −2179. MS (ESI, Fig. S5.2) [M-H]^+^: 657.1 (M = [{*cis-*Pt(NH_3_)_2_}_2_(µ-OH)(µ-5-(butyryloxy)methyl tetrazolato-*N2,N3*)]^2+^)

*[{cis-Pt(NH_3_)_2_}_2_(µ-OH)(µ-5-hydroxymethyltetrazolato-N2,N3)](NO_3_)_2_* (**9**)

Yield: 28 mg (15.0%). ^1^H NMR (400 MHz, D_2_O, Fig. S1.3): *δ* = 4.89 (s, 2H). ^13^C NMR (151 MHz, D_2_O, Fig. S2.3): *δ* = 57.4, 166.9. ^195^Pt NMR (129 MHz, D_2_O, Fig. S4.3): *δ* = −2181. MS (ESI, Fig. S5.2) [M-H]^+^: 573.1 (M = [{*cis-*Pt(NH_3_)_2_}_2_(µ-OH)(µ-5-hydroxymethyltetrazolato-*N2,N3*)]^2+^)

*[{cis-Pt(NH_3_)_2_}_2_(µ-OH)(µ-5-hydroxyethyltetrazolato-N2,N3)](NO_3_)_2_* (**10**)

Yield: 30 mg (18.0%). ^1^H NMR (400 MHz, D_2_O, Fig. S1.3): *δ* = 3.13 (t, 3H, *J* = 6.8 Hz), 3.97 (t, 2H, *J* = 6.8 Hz). ^13^C NMR (151 MHz, D_2_O, Fig. S2.3): *δ* = 30.7, 62.1, 166.2. ^195^Pt NMR (129 MHz, D_2_O, Fig. S4.3): *δ* = −2178. MS (ESI, Fig. S5.2) [M-H]^+^: 587.1 (M = [{*cis-*Pt(NH_3_)_2_}_2_(µ-OH)(µ-5-hydroxyethyltetrazolato-*N2,N3*)]^2+^)

*[{cis-Pt(NH_3_)_2_}_2_(µ-OH)(µ-5-mono-fluoromethyltetrazolato-N2,N3)](NO_3_)_2_* (**11**)

Yield: 320 mg (28.2%). ^1^H NMR (400 MHz, D_2_O, Fig. S1.3): *δ* = 5.67 (d, 2H, ^2^*J*_HF_ = 47 Hz). ^13^C NMR (151 MHz, D_2_O, Fig. S2.3): *δ* = 77.6 (d, ^1^*J*_CF_ = 164 Hz), 163.2. ^19^F NMR (564 MHz, D_2_O, Fig. S3): *δ* = −215.1 (t, 1F, ^2^*J*_FH_ = 48 Hz). ^195^Pt NMR (129 MHz, D_2_O, Fig. S4.3): *δ* = −2183. MS (ESI, Fig. S5.3) [M-H]^+^: 575.0 (M = [{*cis-*Pt(NH_3_)_2_}_2_(µ-OH)(µ-5-monofluoromethyltrazolato-*N2,N3*)]^2+^)

*[{cis-Pt(NH_3_)_2_}_2_(µ-OH)(µ-5-difluoromethyltetrazolato-N2,N3)](NO_3_)_2_* (**12**)

Yield: 262 mg (22.5%). ^1^H NMR (400 MHz, D_2_O, Fig. S1.4): *δ* = 7.15 (t, 1H, ^2^*J*_HF_ = 53 Hz). ^13^C NMR (151 MHz, D_2_O, Fig. S2.4): *δ* = 110.8 (t, ^1^*J*_CF_ = 238 Hz), 161.7 (d, ^2^*J*_CF_ = 28.1 Hz). ^19^F NMR (564 MHz, D_2_O, Fig. S3): *δ* = −116.8 (d, 2F, ^2^*J*_FH_ = 54 Hz). ^195^Pt NMR (129 MHz, D_2_O, Fig. S4.4): *δ* = −2184. MS (ESI, Fig. S5.3) [M-H]^+^: 593.0 (M = [{*cis-*Pt(NH_3_)_2_}_2_(µ-OH)(µ-5-difluoromethyltrazolato-*N2,N3*)]^2+^)

*[{cis-Pt(NH_3_)_2_}_2_(µ-OH)(µ-5-trifluoromethyl tetrazolato-N2,N3)](NO_3_)_2_* (**13**)

Yield: 259 mg (21.7%). ^13^C NMR (151 MHz, D_2_O, Fig. S2.4): *δ* = 121.5 (q, ^1^*J*_CF_ = 269 Hz), 158.8 (d, ^2^*J*_CF_ = 42 Hz). ^19^F NMR (564 MHz, D_2_O, Fig. S3): *δ* = 62.7 (s, 3F). ^195^Pt NMR (129 MHz, D_2_O, Fig. S4.4): *δ* = −2184. MS (ESI, Fig. S5.3) [M-H]^+^: 611.0 (M = [{*cis-*Pt(NH_3_)_2_}_2_(µ-OH)(µ-5-trifluoromethyltrazolato-*N2,N3*)]^2+^)

*[{cis-Pt(NH_3_)_2_}_2_(µ-OH)(µ-5-cyclohexyltetrazolato-N2,N3)](NO_3_)_2_* (**14**)

Yield: 430 mg (35.3%). ^1^H NMR (600 MHz, CD_3_OD, Fig. S1.4): *δ* = 1.29 (m, 1H), 1.41 (m, 2H), 1.55 (m, 2H), 1.72 (m, 1H), 1.80 (m, 2H), 1.98 (m, 2H), 2.98 (1H, m). ^13^C NMR (CD_3_OD, Fig. S2.4): *δ* = 28.2, 34.1, 37.7, 172.6. ^195^Pt NMR (CD_3_OD, Fig. S4.4): *δ* = −2172. MS (ESI, Fig. S5.3) [M-H]^+^: 625.1 (M = [{*cis-*Pt(NH_3_)_2_}_2_(µ-OH)(µ-5-cyclohexyltetrazolato-*N2,N3*)]^2+^)

*[{cis-Pt(NH_3_)_2_}_2_(µ-OH)(µ-5-(adamantan-1-yl)tetrazolato-N2,N3)](NO_3_)_2_* (**15**)

Yield: 364 mg (28.0%). ^1^H NMR (600 MHz, CD_3_OD, Fig. S1.4): *δ* = 1.76-1.84 (m, 6H), 2.01 (m, 6H), 2.07 (m, 3H). ^13^C NMR (CD_3_OD, Fig. S2.4): *δ* = 30.7, 36.2, 38.7, 43.7, 175.8. ^195^Pt NMR (D_2_O, Fig. S4.4): *δ* = −2175. MS (ESI, Fig. S5.3) [M-H]^+^: 677.1 (M = [{*cis-*Pt(NH_3_)_2_}_2_(µ-OH)(µ-5-(adamantan-1-yl)tetrazolato-*N2,N3*)]^2+^)

#### 2.2.2 Synthesis of 1H-Tetrazole derivatives

NaN_3_ (975 mg, 15 mmol) and Et_3_N^.^HCl (2065 mg, 15 mmol) were added to a solution of the nitrile (5 mmol) in PhNO_2_ (10 mL). The reaction mixture was exposed to MW irradiation at 100°C for 2 h. The reaction mixture treated with EtOAc (100 mL), and extracted with 4% aq. NaOH (50 mL × 3). The combined aqueous layers were washed with EtOAc, acidified with 6*N* HCl, and extracted with EtOAc (100 mL × 2). The combined organic layers were dried over Na_2_SO_4_, filtrated, and evaporated to afford a crude residue that was purified by using column chromatography on silica gel with EtOAc [15]. For the synthesis of **SH40**, to a solution of 2-fluoroacetonitrile (885 mg, 15 mmol) in DMF (10 mL) NaN_3_ (1950 mg, 30 mmol) and Et_3_N·HCl (4120 mg, 30 mmol) were added. After stirring for 3 h at 80 °C, the reaction mixture was dissolved in EtOAc (150 mL). The organic layer was washed with 2*N* HCl (50 mL × 4) and then brine, and then dried over Na_2_SO_4_, filtered, and concentrated to afford a crude residue that was recrystallized from hexane to give white needles (m.p. 79–80 °C). For the synthesis of **SH41**,to a solution of 2,2-difluoroacetonitrile (1155 mg, 15 mmol) in DMF (10 mL) NaN_3_ (1950 mg, 30 mmol) and Et_3_N·HCl (4120 mg, 30 mmol) were added. After stirring for 20 h at r.t., the reaction mixture was dissolved in EtOAc (150 mL). The organic layer was washed with 2*N* HCl (50 mL × 4), brine, and then dried over Na_2_SO_4_, filtered, and concentrated to afford a crude residue that was recrystallized from hexane to give prisms of compound (m.p. 98–99 °C).

*(1H-tetrazol-5-yl)methyl acetate (* ***SH23*** *, 5-[(acetoxy)methyl]-1H-tetrazole)* 

Yield: 450 mg (63%). ^1^H NMR (300 MHz, CDCl_3_): *δ* = 2.17 (s, 3H), 5.55 (s, 2H). ^13^C NMR (75 MHz, CDCl_3_): *δ* = 20.5, 55.0, 153.1, 171.3.

*(1H-tetrazol-5-yl)methyl propionate (* ***SH22*** *, 5-[(propionyloxy)methyl]-1H-tetrazole)* 

Yield: 385 mg (49%). ^1^H NMR (400 MHz, CDCl_3_): *δ* = 1.12 (t, 3H, *J* = 7.6 Hz), 2.44 (q, 2H. *J* = 7.6 Hz), 5.59 (s, 2H). ^13^C-NMR (100 MHz, CDCl_3_): *δ* = 8.6, 27.0, 54.8, 153.2, 174.5. HRMS (EI): *m*/*z* [M^+^] calcd for C_5_H_9_N_4_O_2_: 157.0725; found: 157.0730.

*(1H-tetrazol-5-yl)methyl butyrate (* ***SH34*** *, 5-[(butyryloxy)methyl]-1H-tetrazole)* 

Yield: 600 mg (70%). ^1^H NMR (400 MHz, CDCl_3_): *δ* = 0.91 (t, 3H, *J* = 7.6 Hz), 1.64 (sx, 2H, *J* = 7.6 Hz), 2.39 (t, 2H, *J* = 7.6 Hz), 5.60 (s, 2H). ^13^C NMR (100 MHz, CDCl_3_): *δ* = 13.4, 18.0, 35.4, 54.7, 153.2, 173.6. HRMS (EI): *m*/*z* [M^+^] calcd for C_6_H_11_N_4_O_2_: 171.0882; found: 171.0879.

*2-(1H-tetrazol-5-yl)ethyl propionate (* ***SH20*** *, 5-[(propionyloxy)ethyl]-1H-tetrazole)* 

Yield: 404 mg (47%). ^1^H NMR (400 MHz, CDCl_3_): *δ* = 1.09 (t, 3H, *J* = 7.2 Hz), 2.33 (q, 2H, *J* = 7.2 Hz), 3.49 (t, 2H, *J* = 6.4 Hz), 4.58 (t, 2H, *J* = 6.4 Hz). ^13^C NMR (75 MHz, CD_3_OD): *δ* = 9.2, 24.4, 28.0, 62.2, 155.4, 175.6. HRMS (EI): *m*/*z* [M^+^] calcd for C_6_H_11_N_4_O_2_: 171.0882; found: 171.0881.

*2-(1H-tetrazol-5-yl)ethyl butyrate (* ***SH33*** *, 5-[(butyryloxy)ethyl]-1H-tetrazole)* 

Yield: 482 mg (52%). ^1^H NMR (400 MHz, CDCl_3_): *δ* = 0.88 (t, 3H, *J* = 7.2 Hz), 1.57 (sx, 2H, *J* = 7.2 Hz), 2.28 (t, 2H, *J* = 7.2 Hz), 3.49 (t, 2H, *J* = 6.4 Hz), 4.58 (t, 2H, *J* = 6.4 Hz). ^13^C NMR (100 MHz, CDCl_3_): *δ* = 13.5, 18.2, 23.8, 35.9, 60.9, 154.2, 174.0. HRMS (EI): *m*/*z* [M^+^] calcd for C_7_H_13_N_4_O_2_: 185.1039; found: 185.1037.

*5-(Fluoromethyl)-1H-tetrazole (**SH40**)* 

Yield: 966 mg (63%). ^1^H NMR (300 MHz, CD_3_OD): *δ* = 5.75 (d, 2H, *J* = 46.8 Hz). ^13^C NMR (75 MHz, CD_3_OD): *δ* = 75.0 (d, *J* = 165.0 Hz), 155.0. ^19^F NMR (282 MHz, CD_3_OD): *δ* = −221.7 (t, *J* = 46.8 Hz). HRMS (EI): *m*/*z* [M^+^] calcd for C_2_H_3_FN_4_: 102.0342; found: 102.0339.

*5-(Difluoromethyl)-1H-tetrazole (**SH41**)* 

Yield: 1419 mg (79%). ^1^H NMR (400 MHz, CD_3_OD): *δ* = 7.24 (t, 1H, *J* = 52.8 Hz). ^13^C-NMR (100 MHz, CD_3_OD): *δ* = 109.5 (t, *J* = 236.0 Hz), 156.6. ^19^F NMR (376 MHz, CD_3_OD): *δ* = −117.2 (d, *J* = 52.8 Hz). HRMS (EI): *m*/*z* [M^+^] calcd for C_2_H_2_F_2_N_4_: 120.0248; found: 120.0243.

## CRediT authorship contribution statement

**Seiji Komeda:** Funding acquisition, Project administration, Supervision, Investigation, Writing – original draft, Writing – review & editing. **Hiroki Yoneyama:** Investigation, Writing – original draft. **Masako Uemura:** Investigation, Writing – original draft. **Takahiro Tsuchiya:** Investigation. **Miyuu Hoshiyama:** Investigation. **Tomoya Sakazaki:** Investigation. **Keiichi Hiramoto:** Investigation, Writing – original draft. **Shinya Harusawa:** Supervision, Writing – review & editing.

## Declaration of Competing Interest

The authors declare that they have no competing interests.

## Appendix Supplementary materials

Image, application 1

Image, application 2

Image, application 3

Image, application 4

Image, application 5
