# Supplementary material for: Data on synthesis and structure–activity relationships of tetrazolato-bridged dinuclear platinum(II) complexes
Source: Data Brief. 2021 Dec 10;40:107697. doi: 10.1016/j.dib.2021.107697 (PMC8713121; doi:10.1016/j.dib.2021.107697)
Supplement: Supplementary file 1 [file mmc1.docx]

Supporting Information

Data on synthesis and structure–activity relationships of tetrazolato-bridged dinuclear platinum(II) complexes.

Seiji Komeda,^1^* Hiroki Yoneyama,^2^ Masako Uemura,^1^ Takahiro Tsuchiya,^1^ Miyuu Hoshiyama,^1^ Tomoya Sakazaki,^1^ Keiichi Hiramoto,^1^ Shinya Harusawa^2^*

*^1^* Faculty of Pharmaceutical Sciences, Suzuka University of Medical Science, Suzuka, Mie 513-8670, Japan

*^2^* Department of Pharmaceutical Organic Chemistry, Osaka Medical and Pharmaceutical University, Takatsuki, Osaka 569-1094, Japan

^1^H NMR


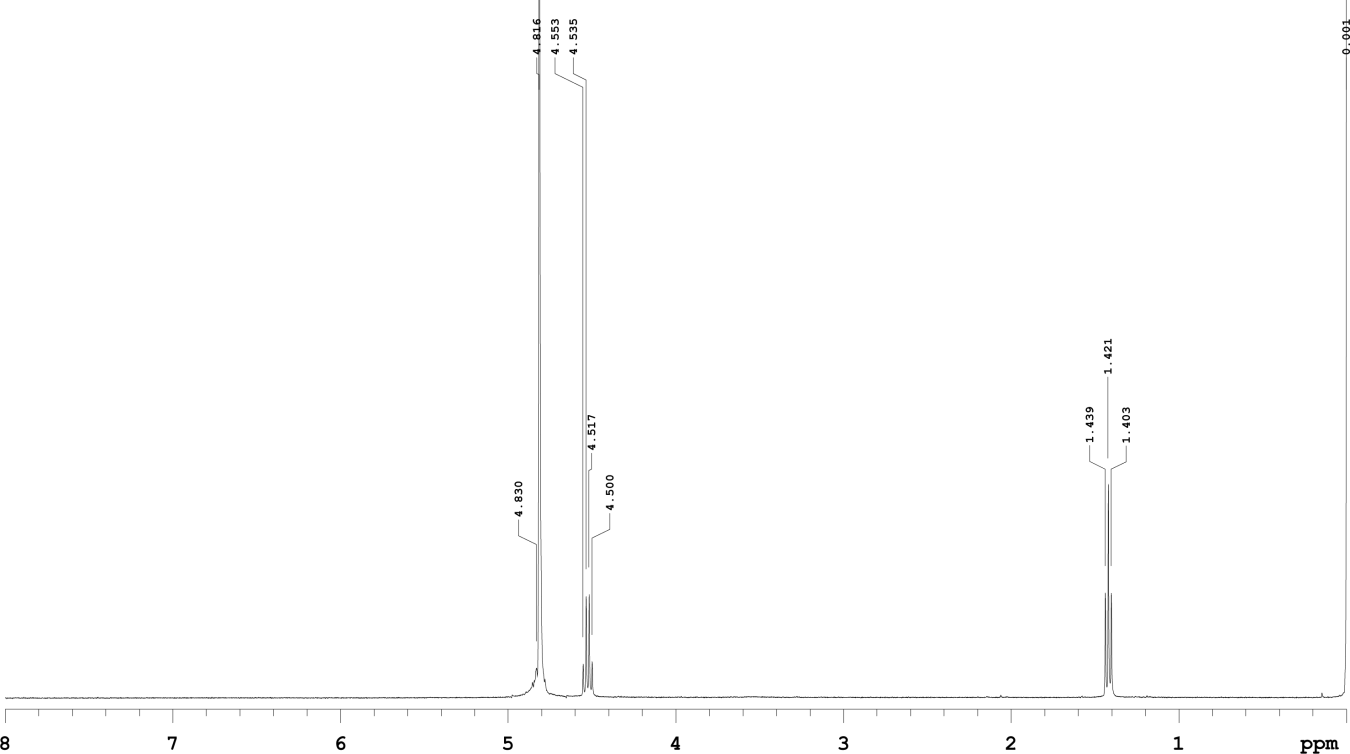


Complex **1**


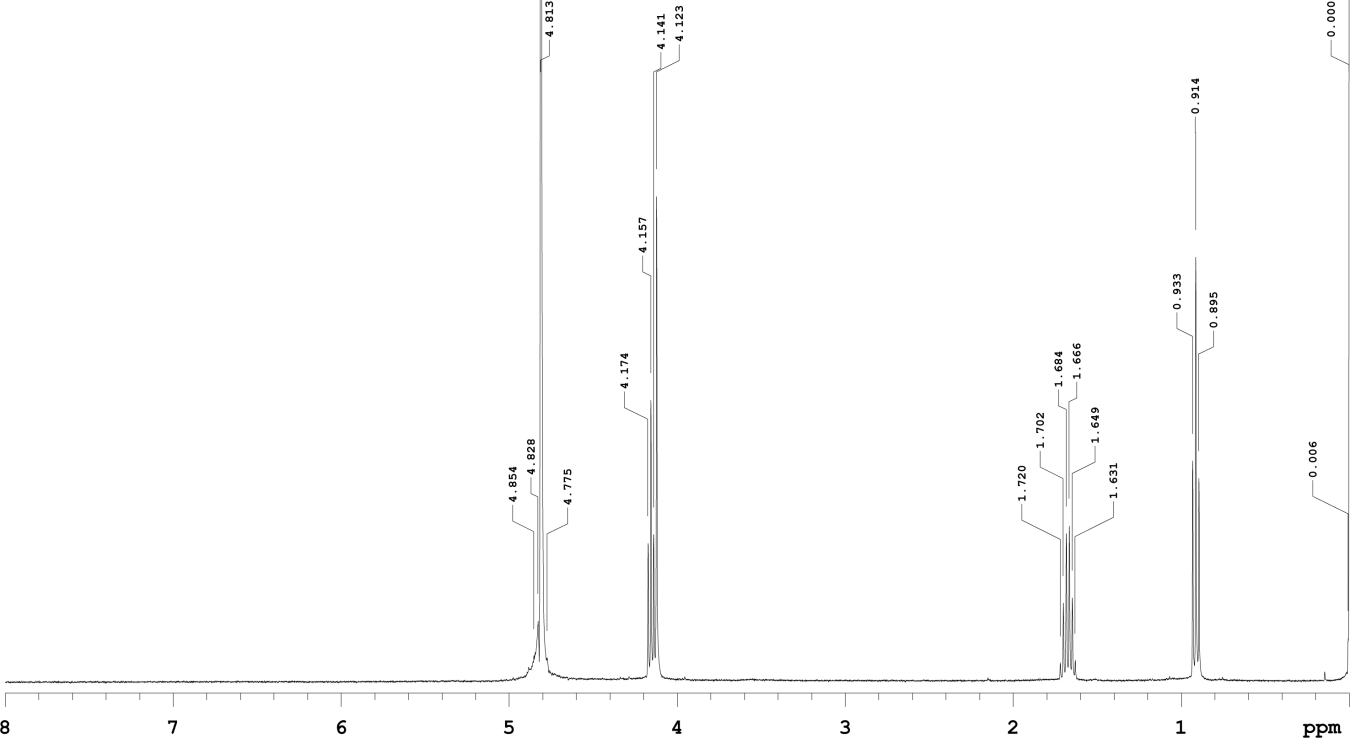


Complex **2**

**Fig. S1.1.** ^1^H NMR spectra of complexes **1** and **2** (D_2_O).


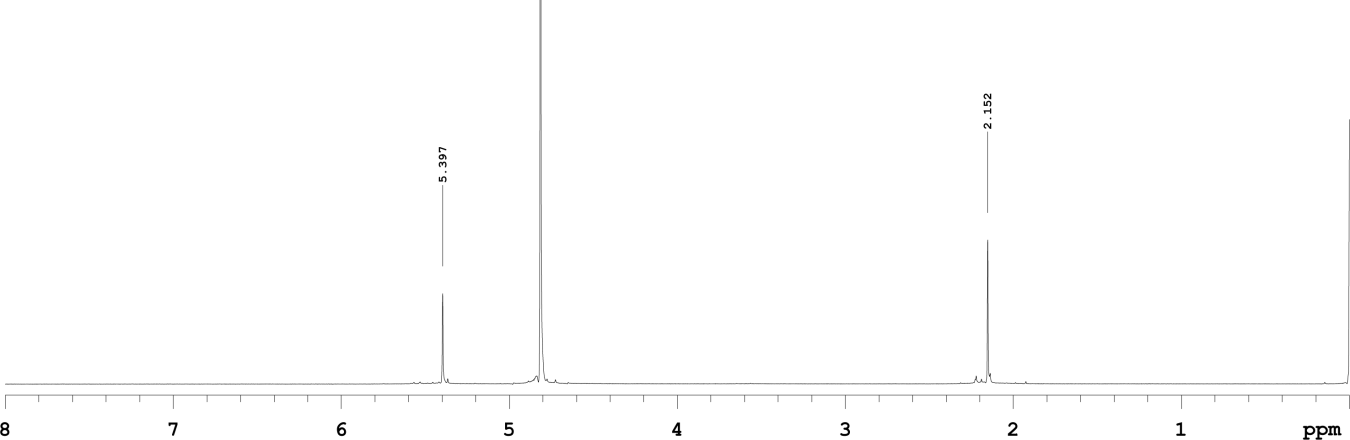


^1^H NMR

Complex **4**


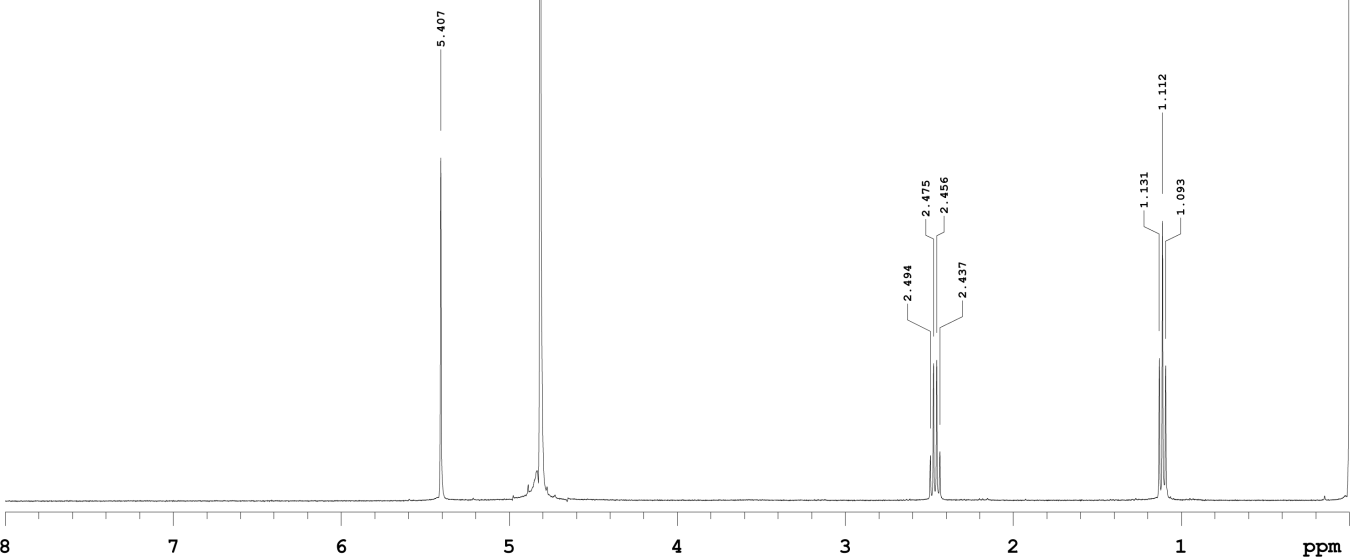


Complex **5**


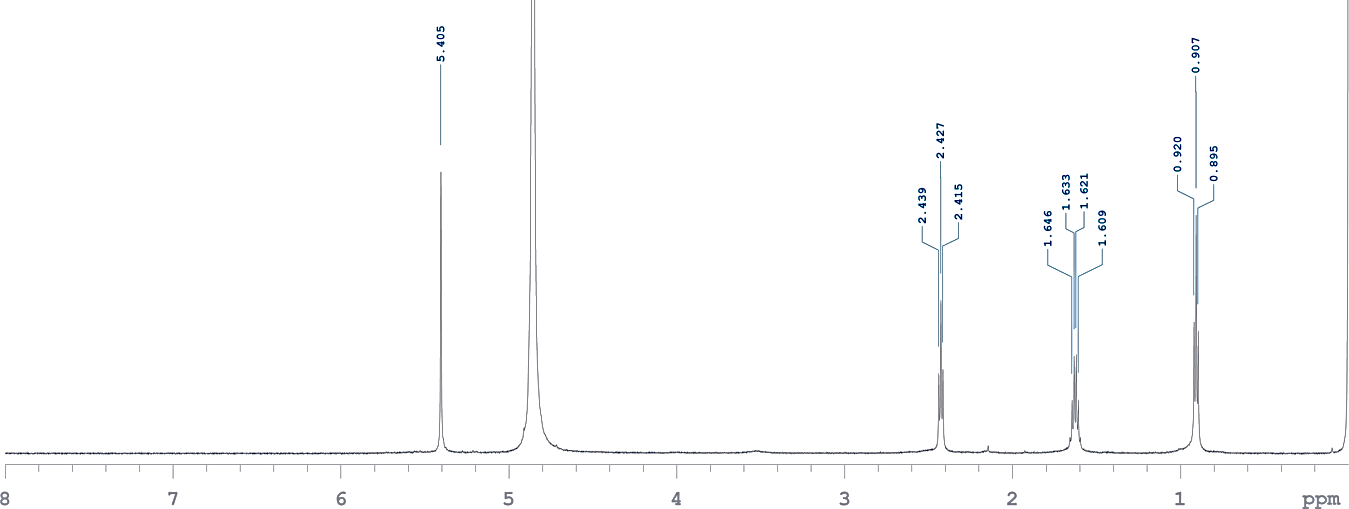


Complex **6**


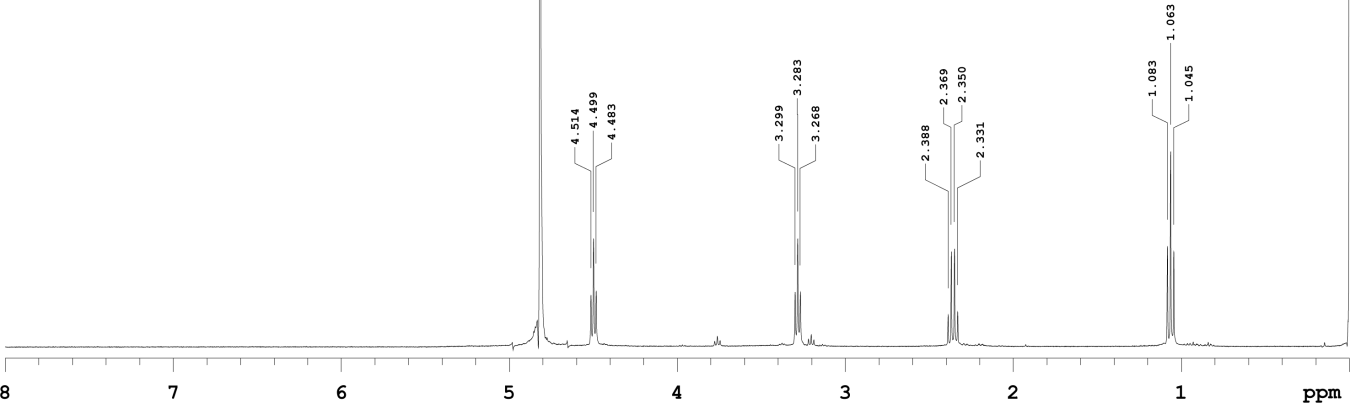


Complex **7**

**Fig. S1.2.** ^1^H NMR spectra of complexes **4**-**7** (D_2_O).


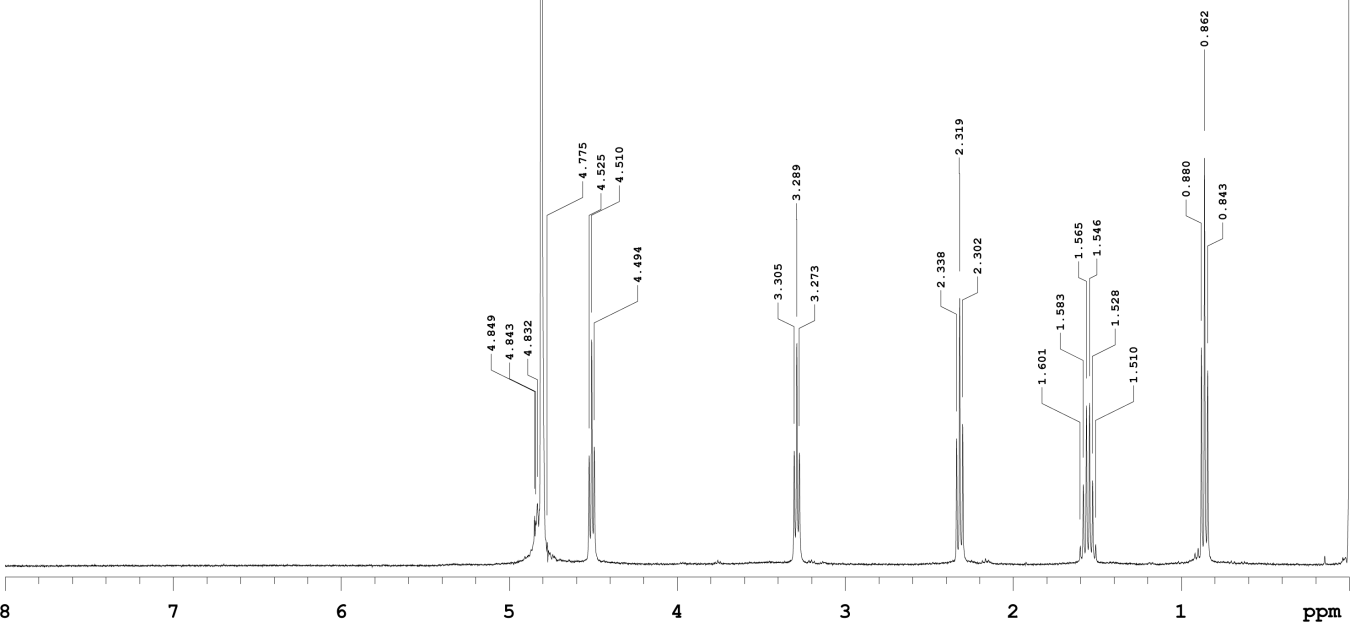


^1^H NMR

Complex **8**


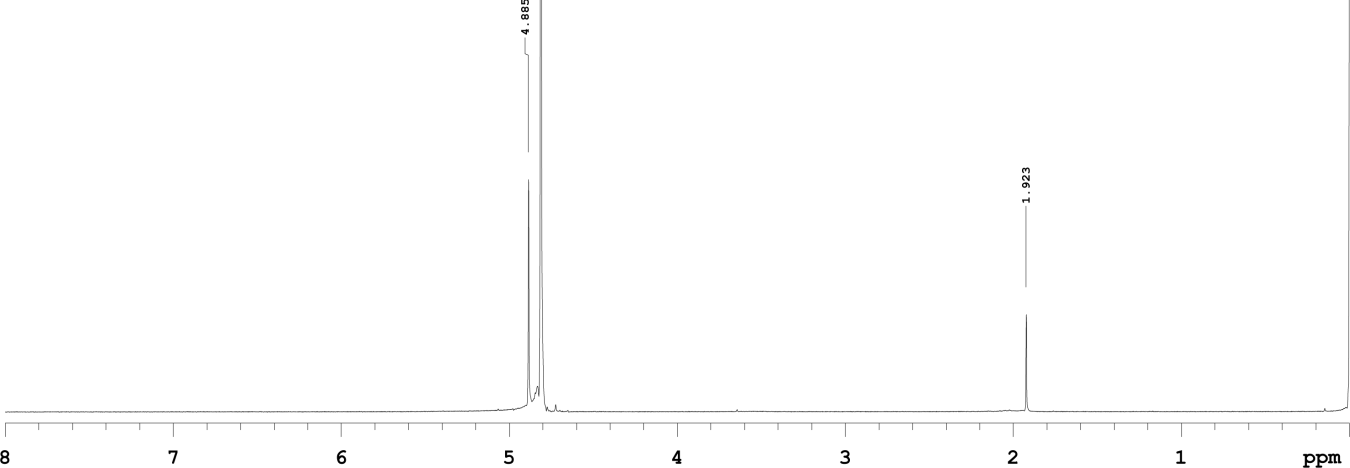


Complex **9**


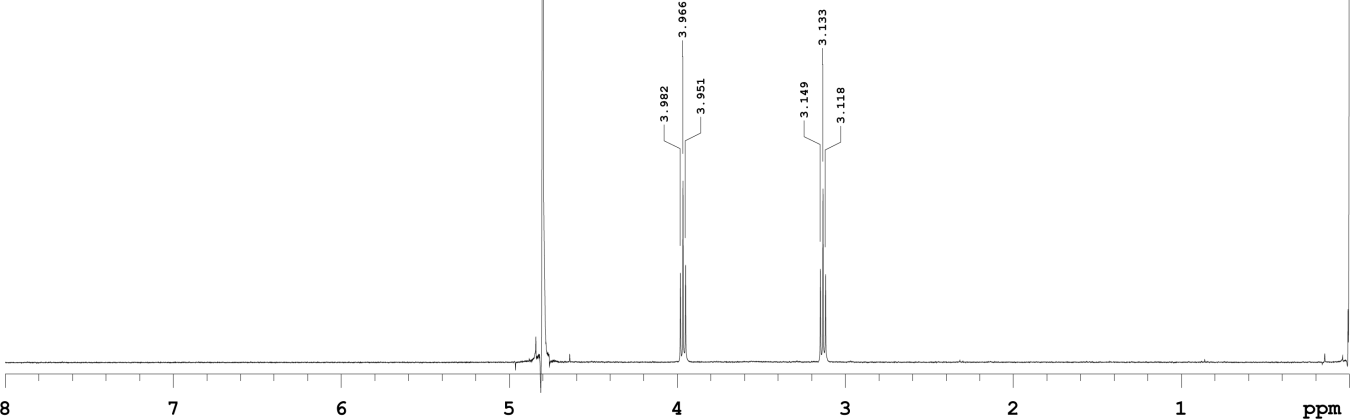


Complex **10**


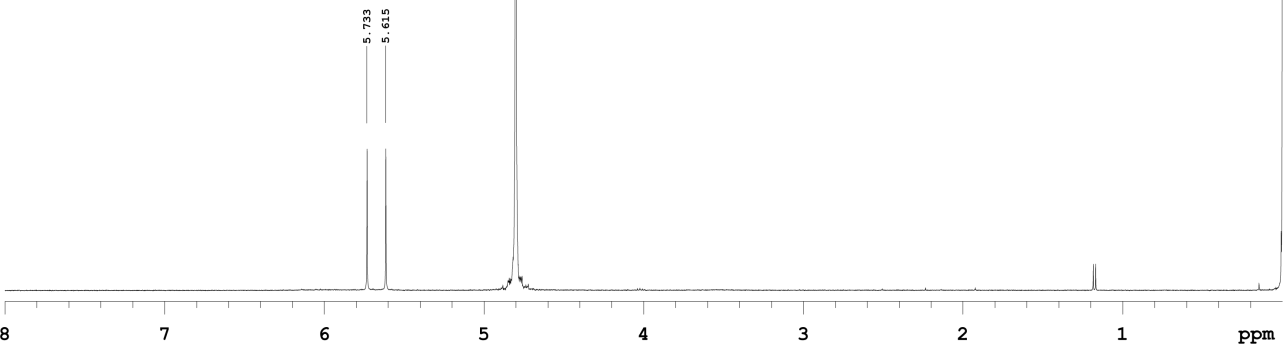


Complex **11**

**Fig. S1.3.** ^1^H NMR spectra of complexes **8-11** (D_2_O).

^1^H NMR


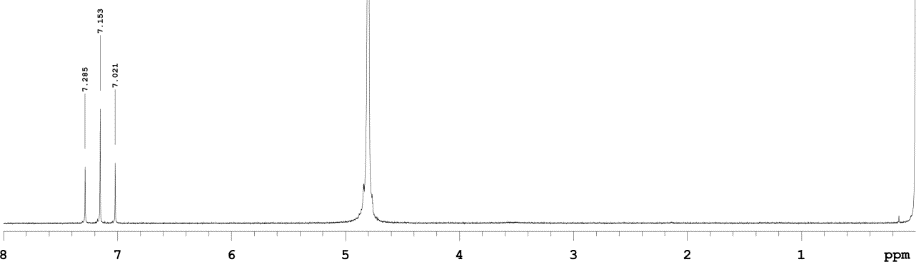


Complex **12**


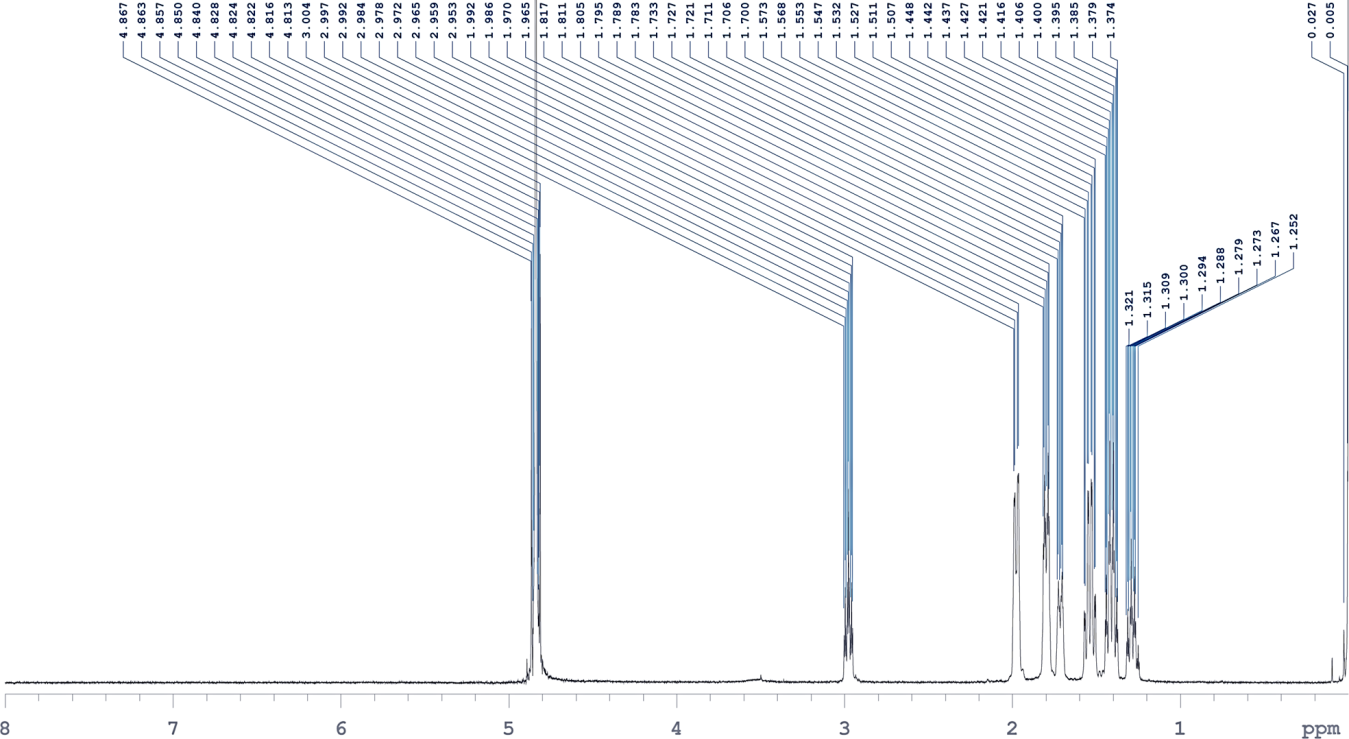


Complex **14**


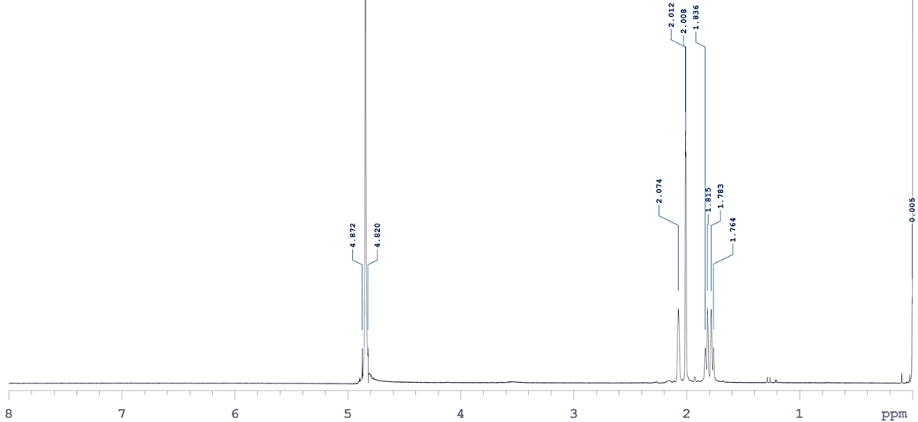


Complex **15**

**Fig. S1.4.** ^1^H NMR spectra of complexes **12, 14** and **15** (D_2_O).

^13^C NMR


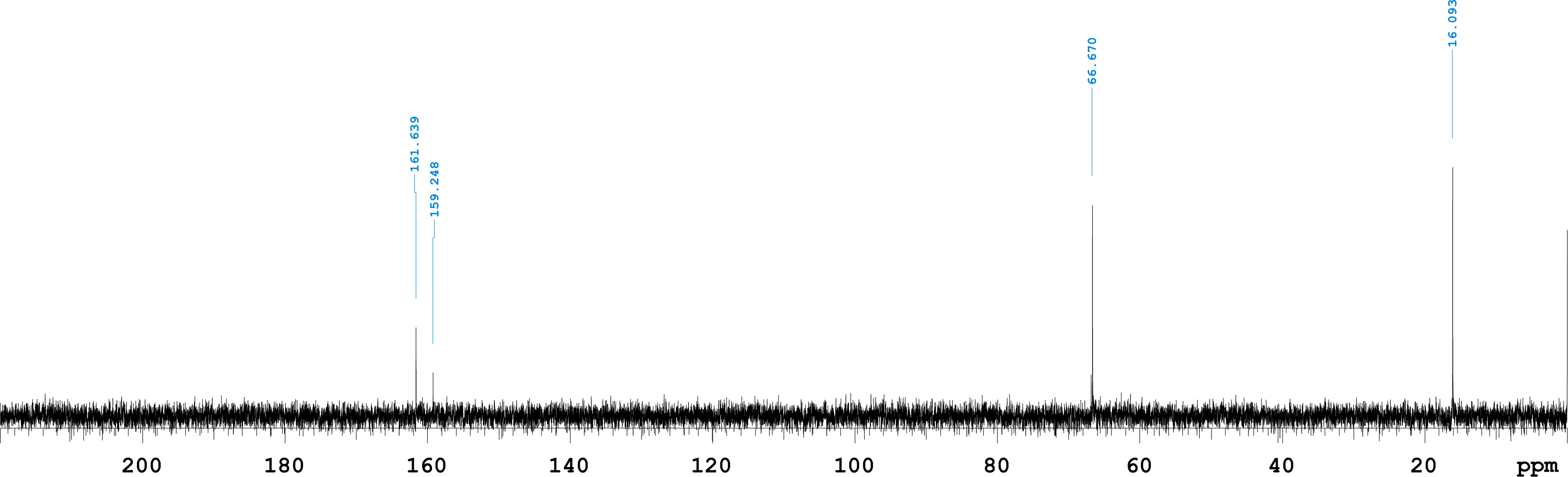


Complex **1**


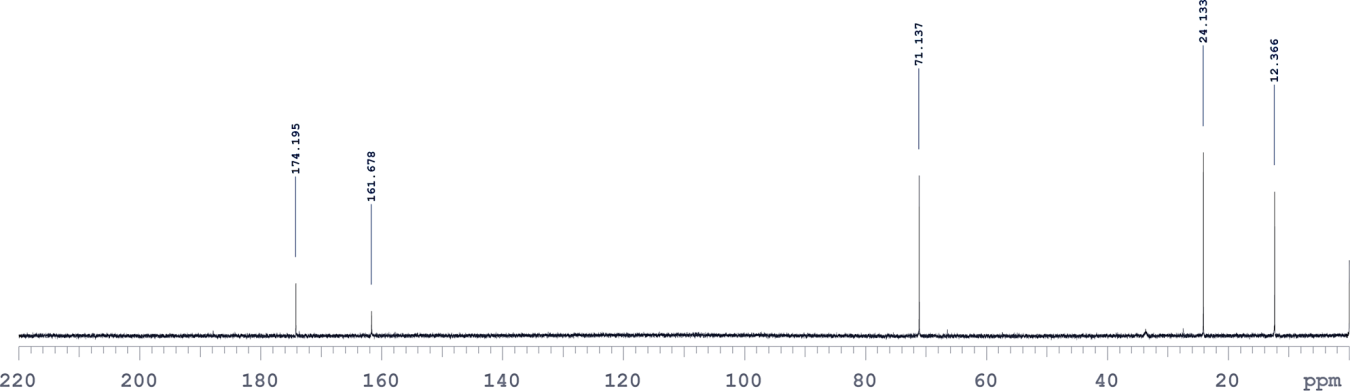


Complex **2**


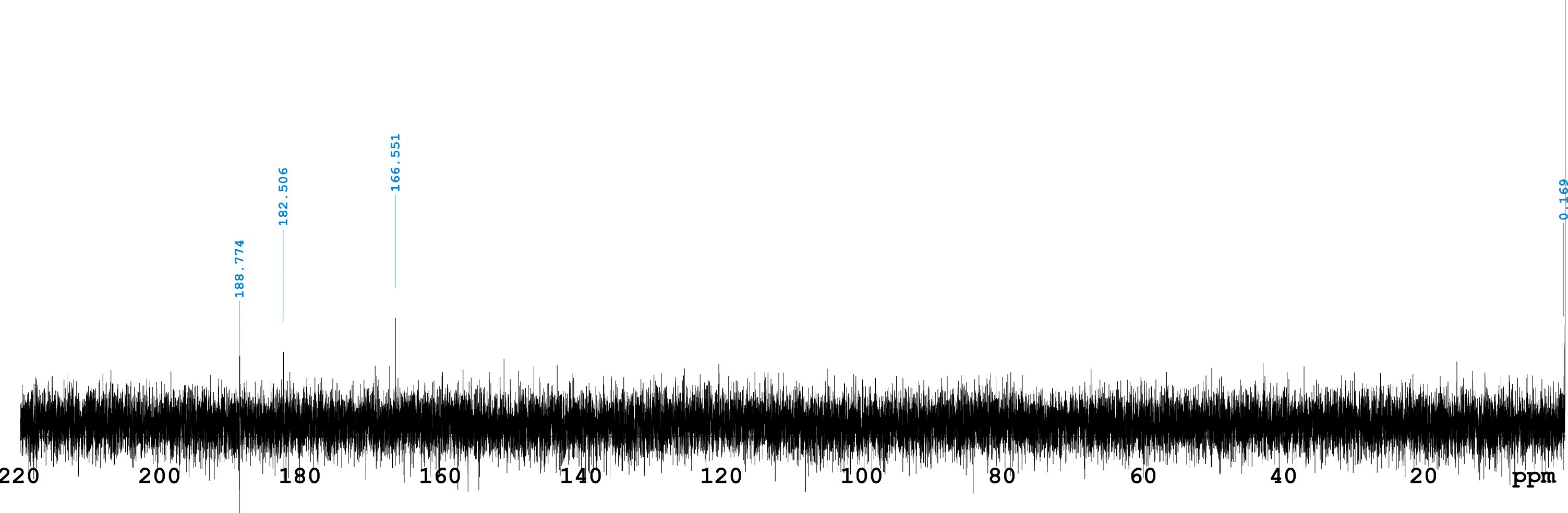


TSP

Complex **3**

**Fig. S2.1.** ^13^C NMR spectra of complexes **1**—**3** (D_2_O).

^13^C NMR


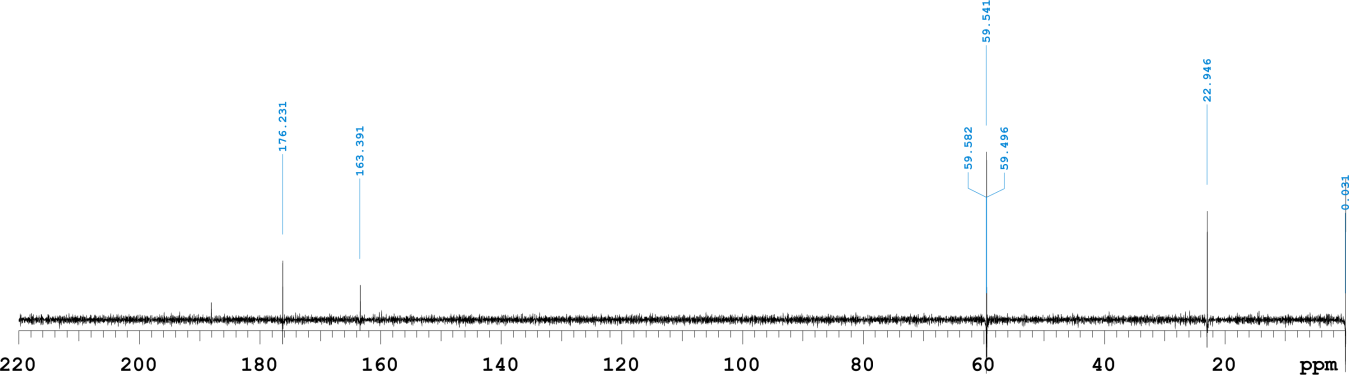


Complex **4**

Complex **7**

TSP

TSP

TSP


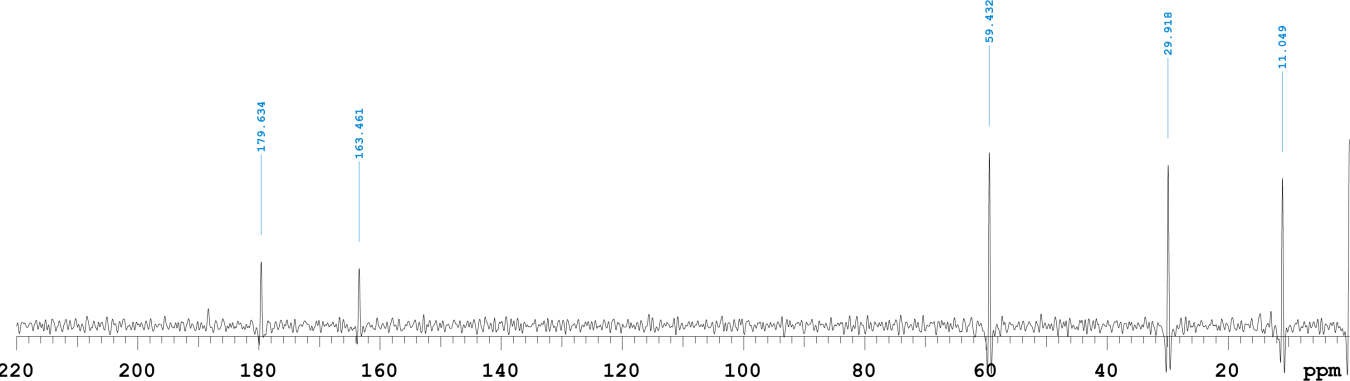


Complex **5**


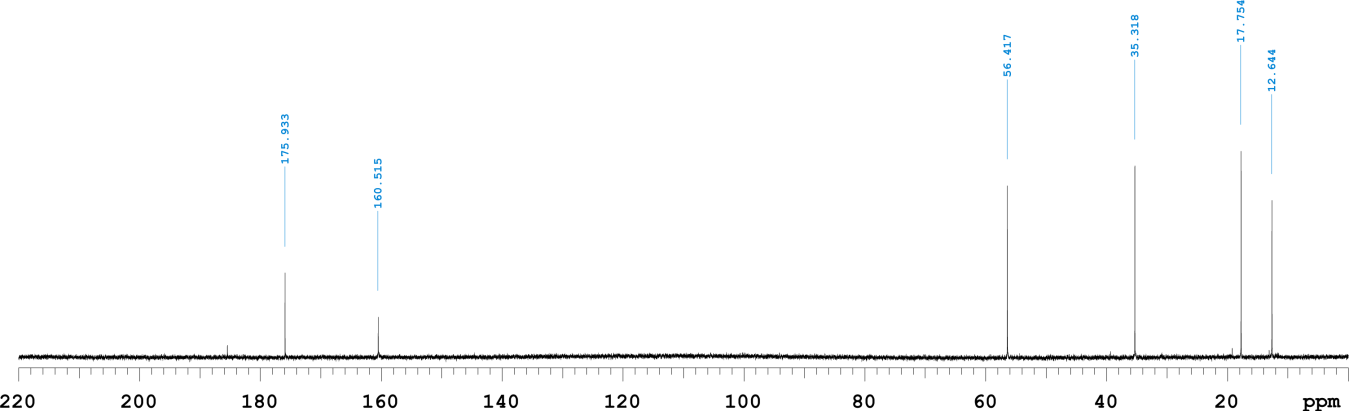


Complex **6**


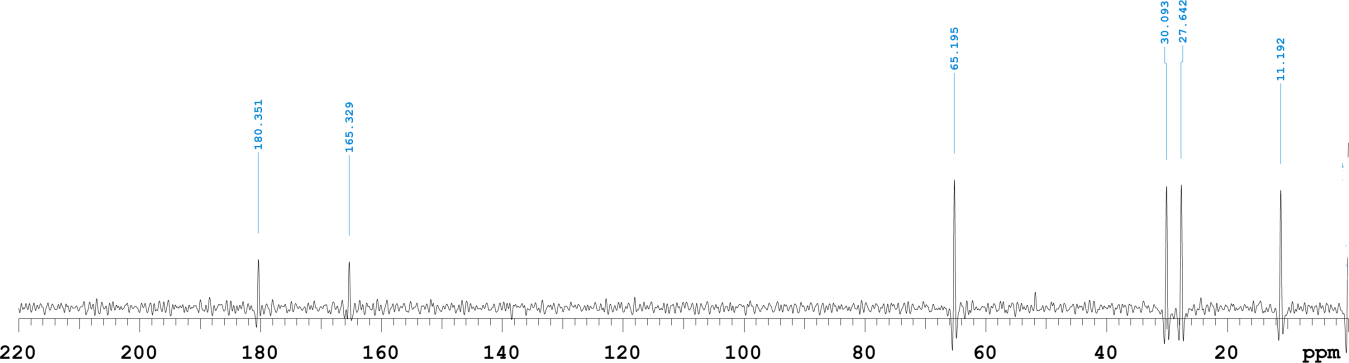


TSP

**Fig. S2.2.** ^13^C NMR spectra of complexes **4-7** (D_2_O).

^13^C NMR


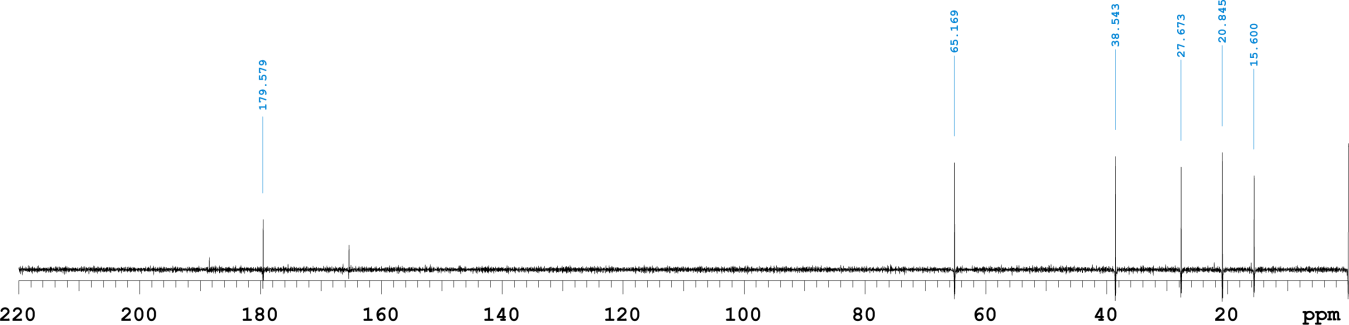


TSP

Complex **8**


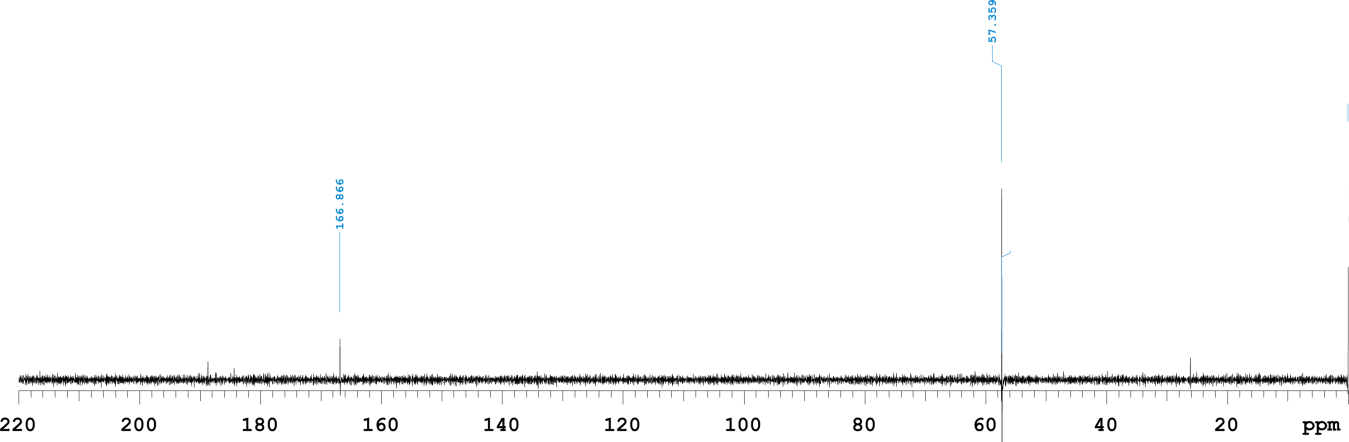


Complex **9**

TSP


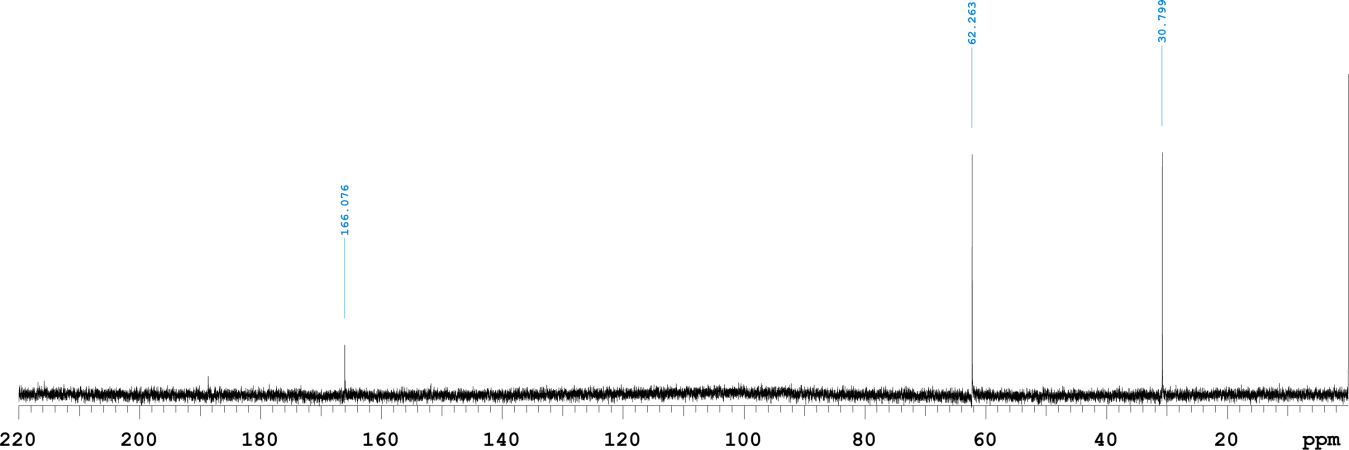


Complex **10**

TSP


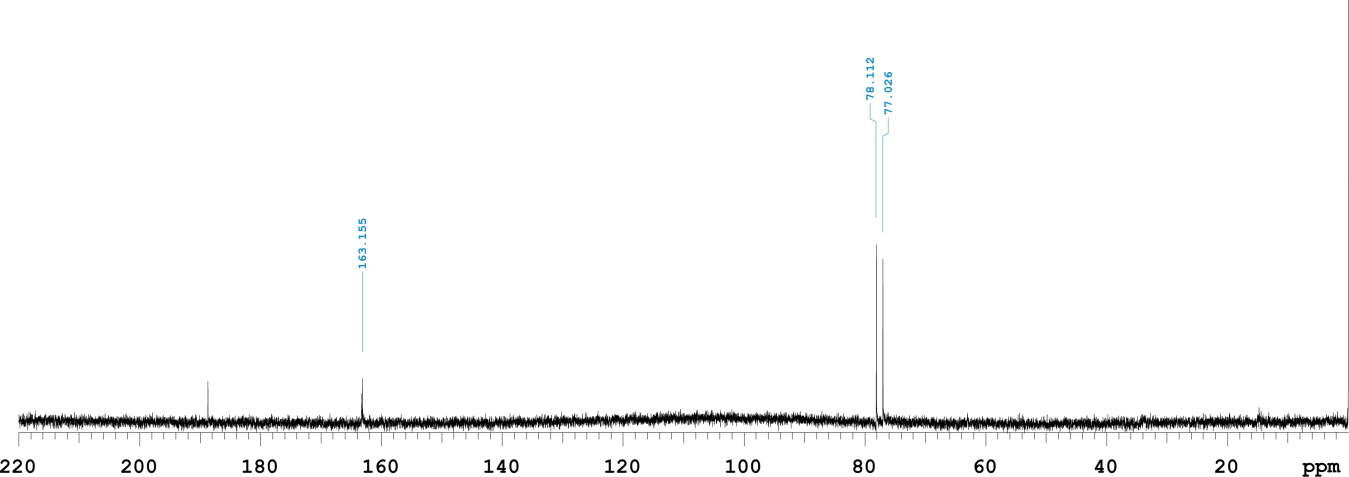


Complex **11**

TSP

**Fig. S2.3.** ^13^C NMR spectra of complexes **8**—**11** (D_2_O).

^13^C NMR


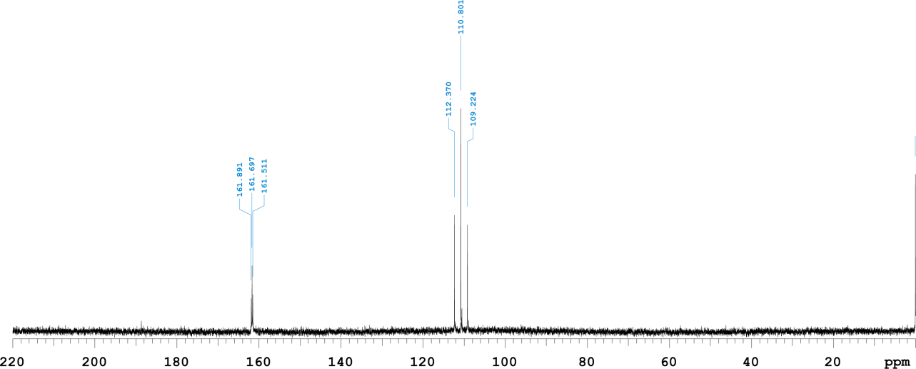


Complex **12**

TSP


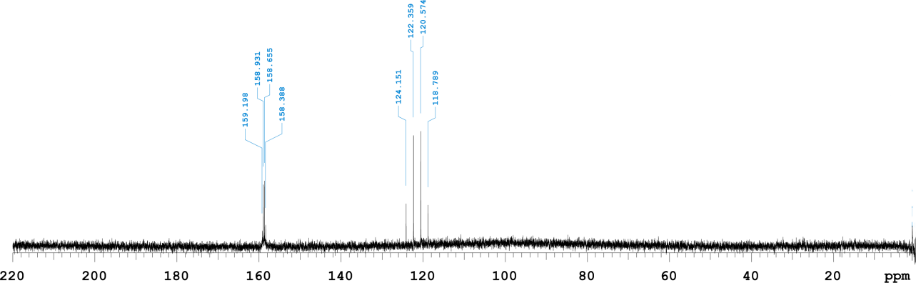


Complex **13**


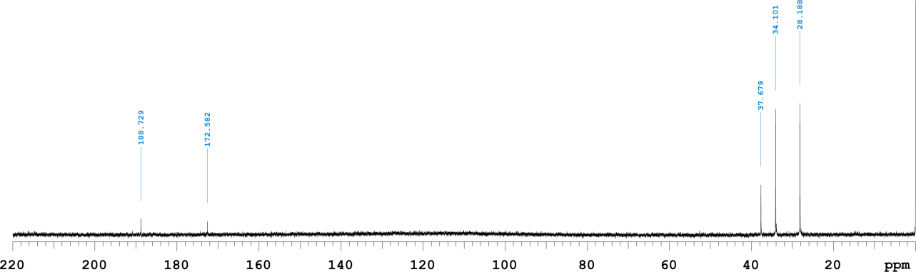


Complex **14**

TSP


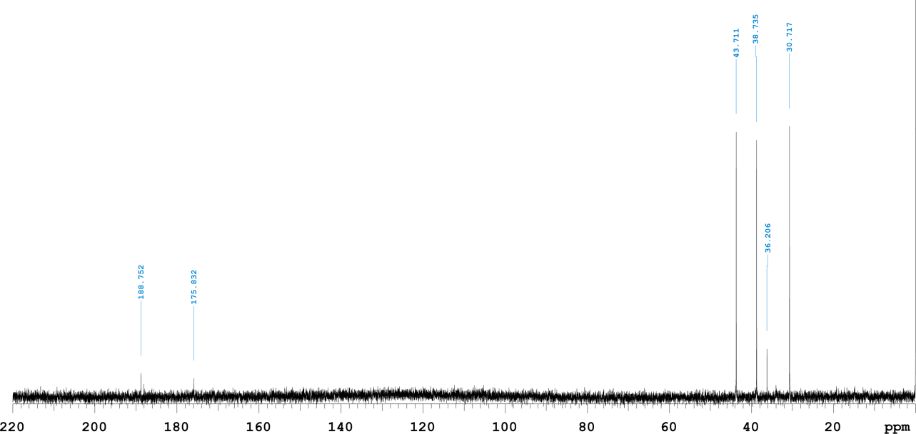


Complex **15**

TSP

**Fig. S2.4.** ^13^C NMR spectra of complexes **12-15** (D_2_O).

^19^F NMR


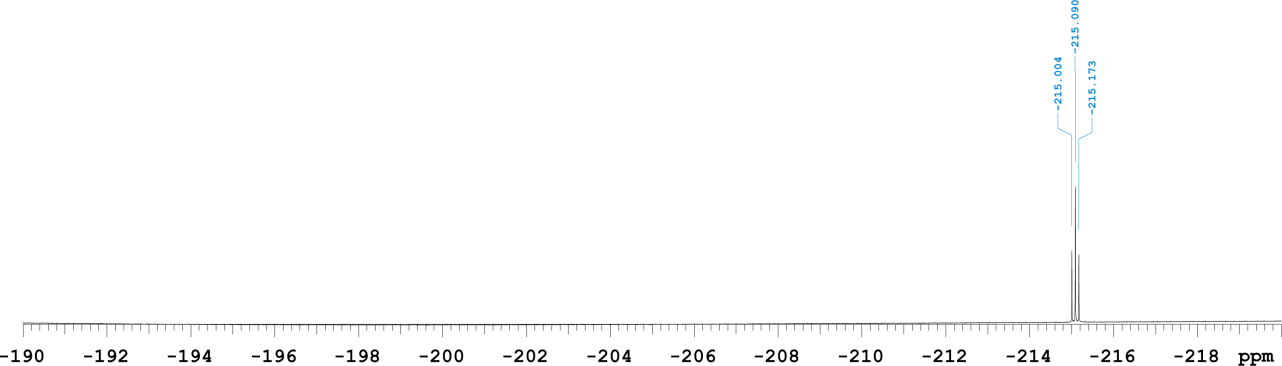


Complex **11**


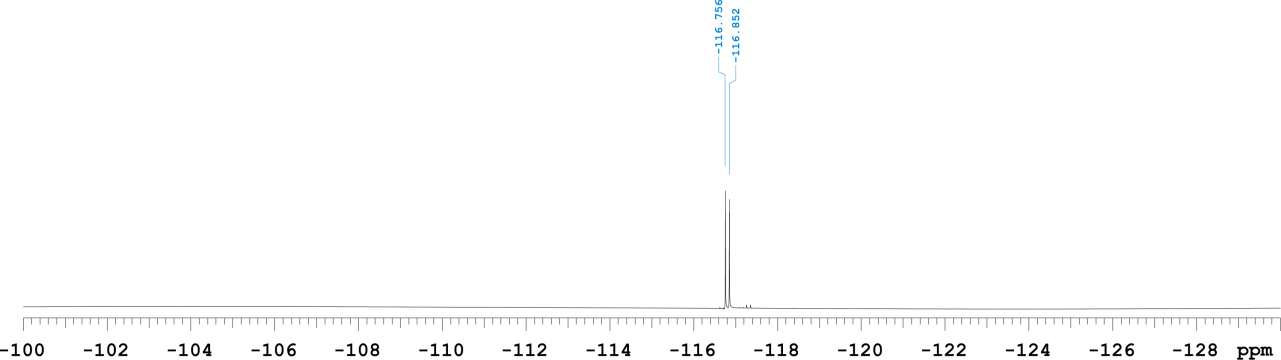


Complex **12**


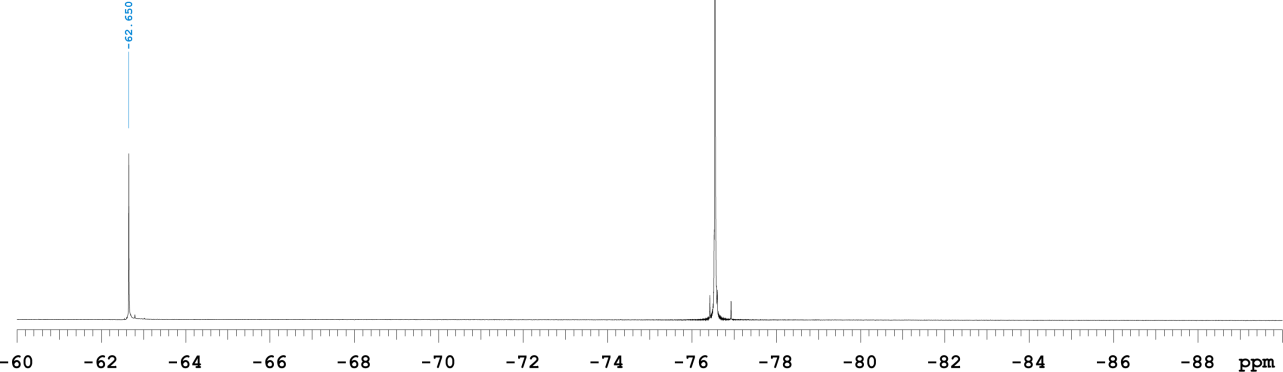


CF_3_COOH

Complex **13**

**Fig. S3.** ^19^F NMR spectra of complexes **11**—**13** (D_2_O).


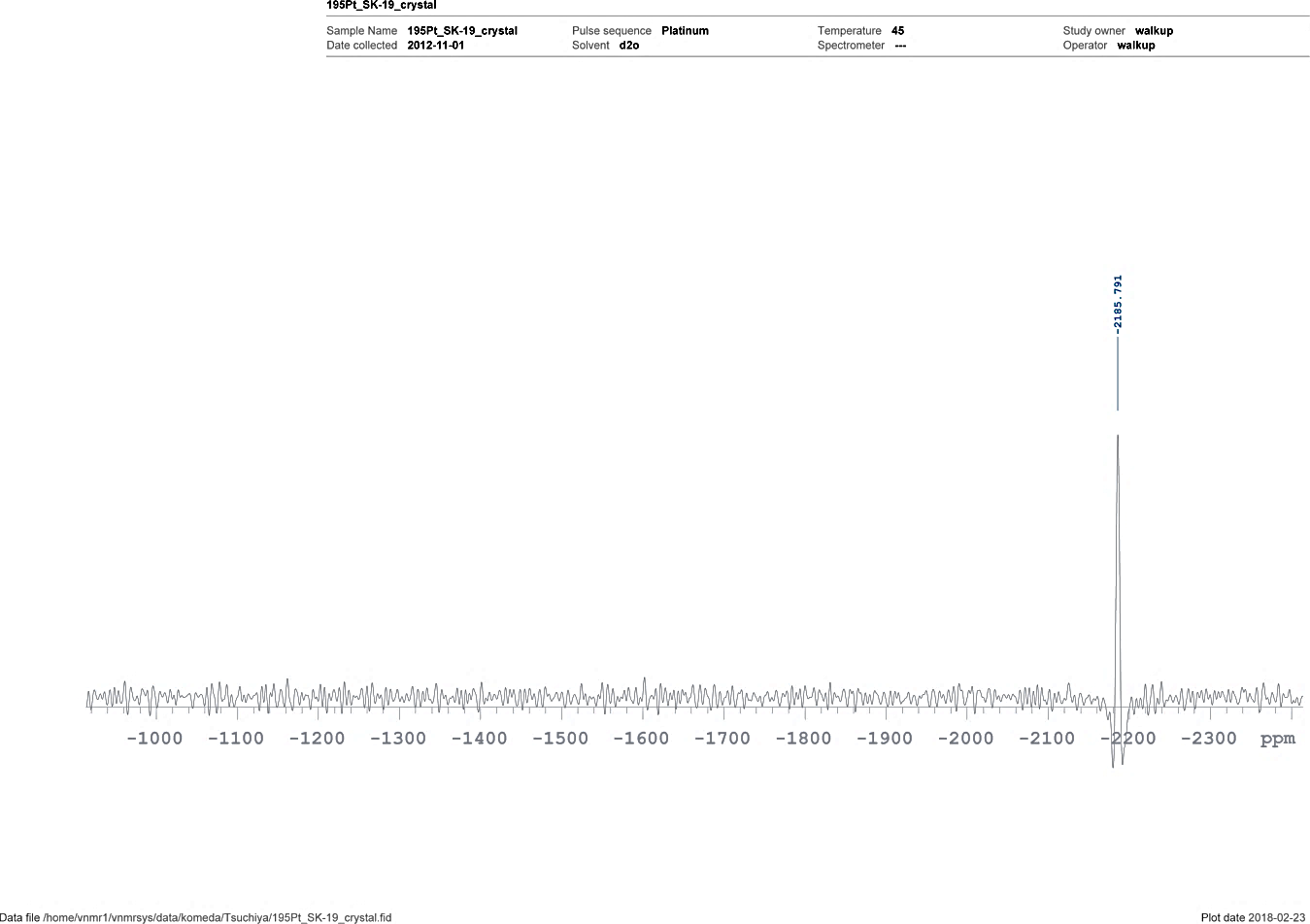


^195^Pt NMR

Complex **1**


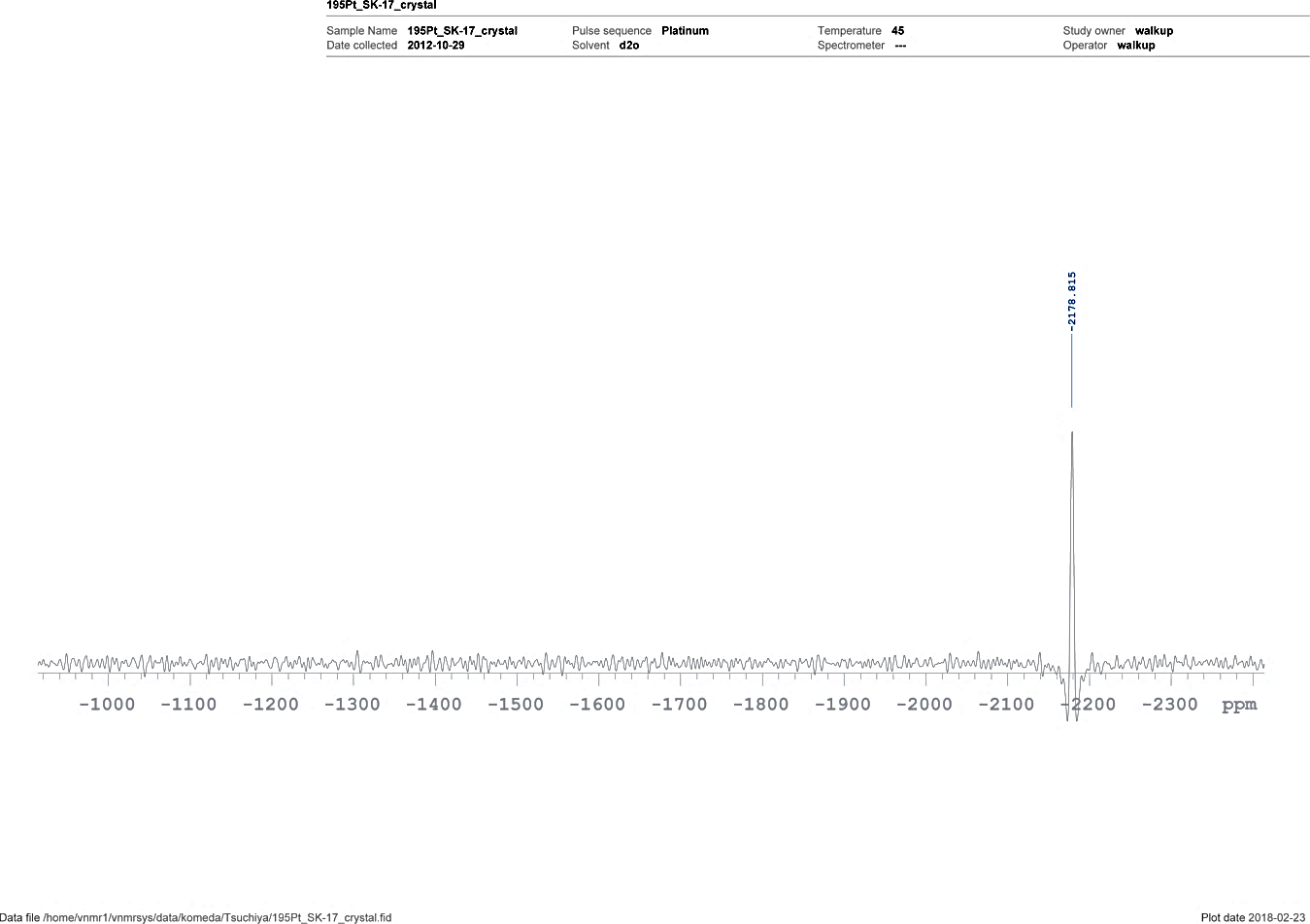


Complex **2**


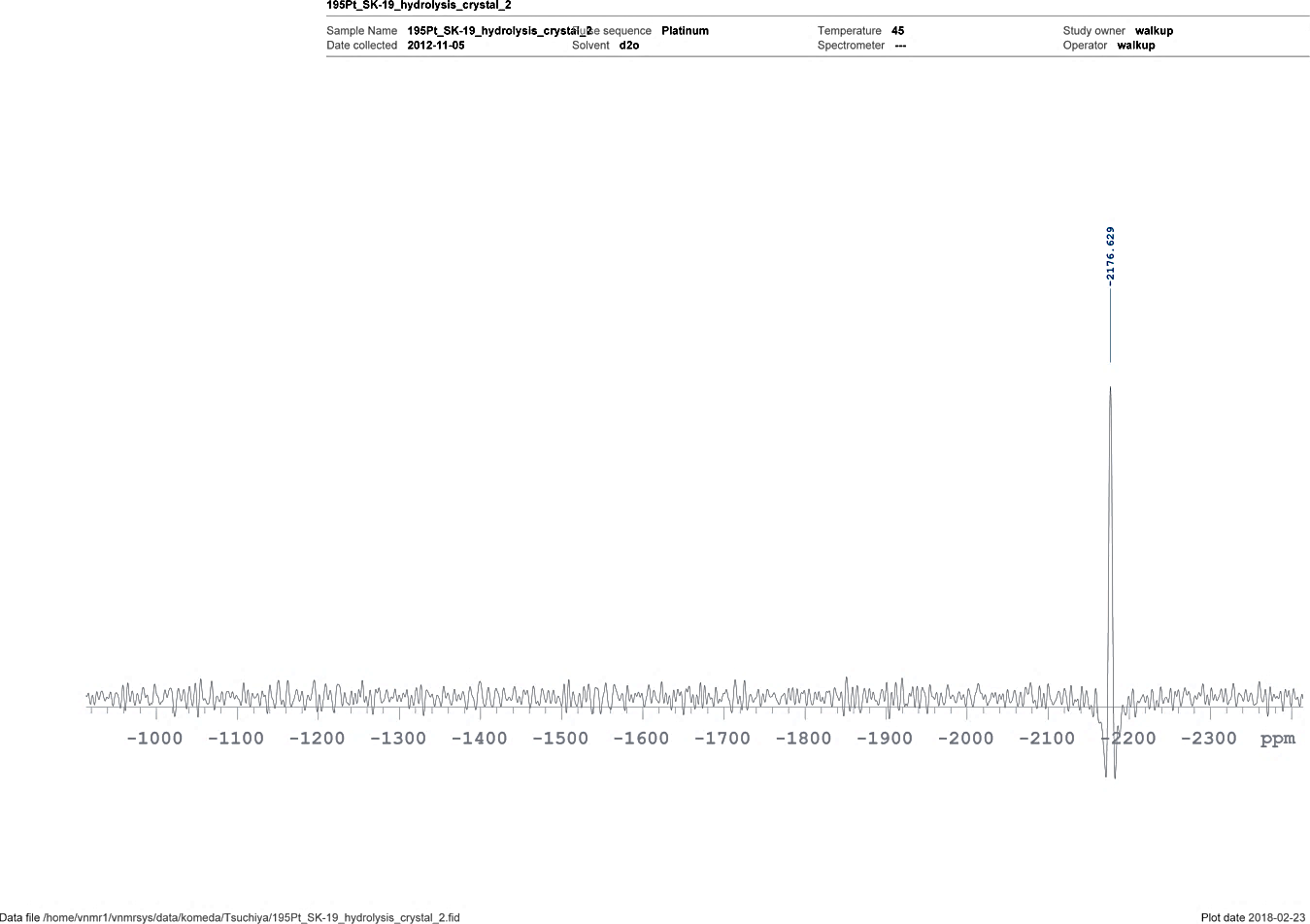


Complex **3**

**Fig. S4.1.** ^195^Pt NMR spectra of complexes **1-3** (D_2_O).


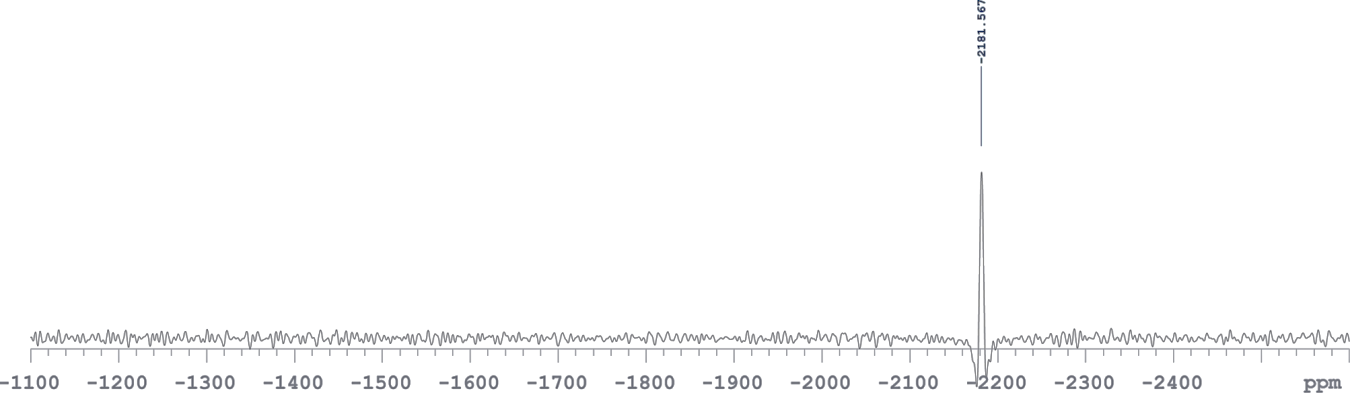


^195^Pt NMR

Complex **4**


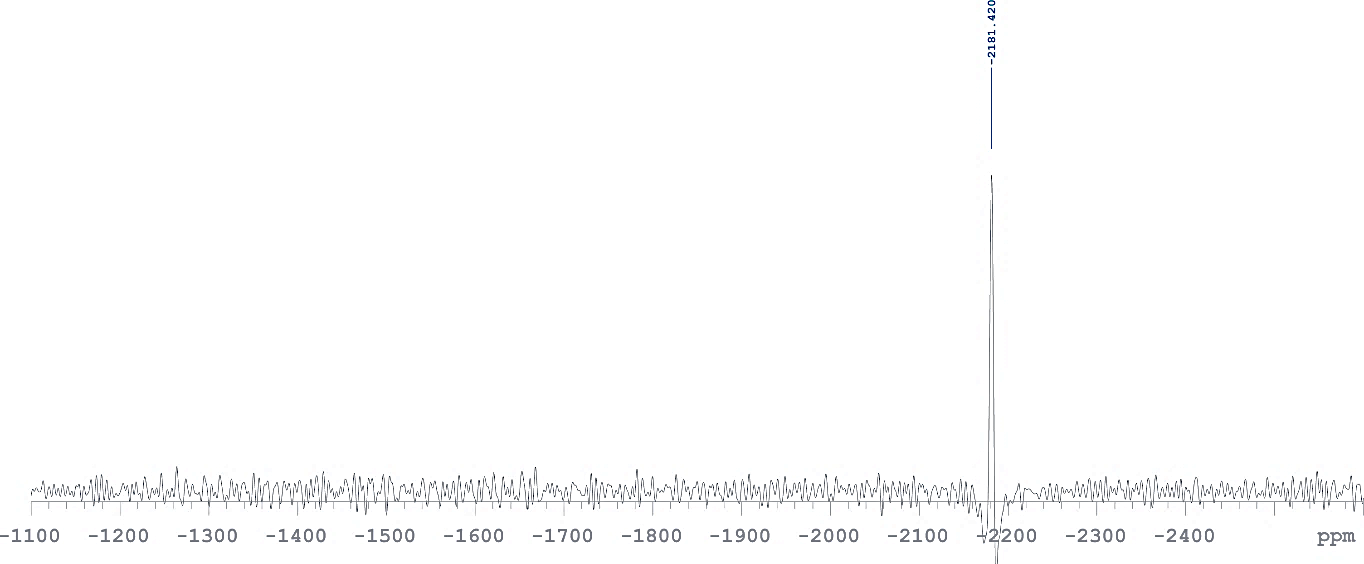


Complex **5**


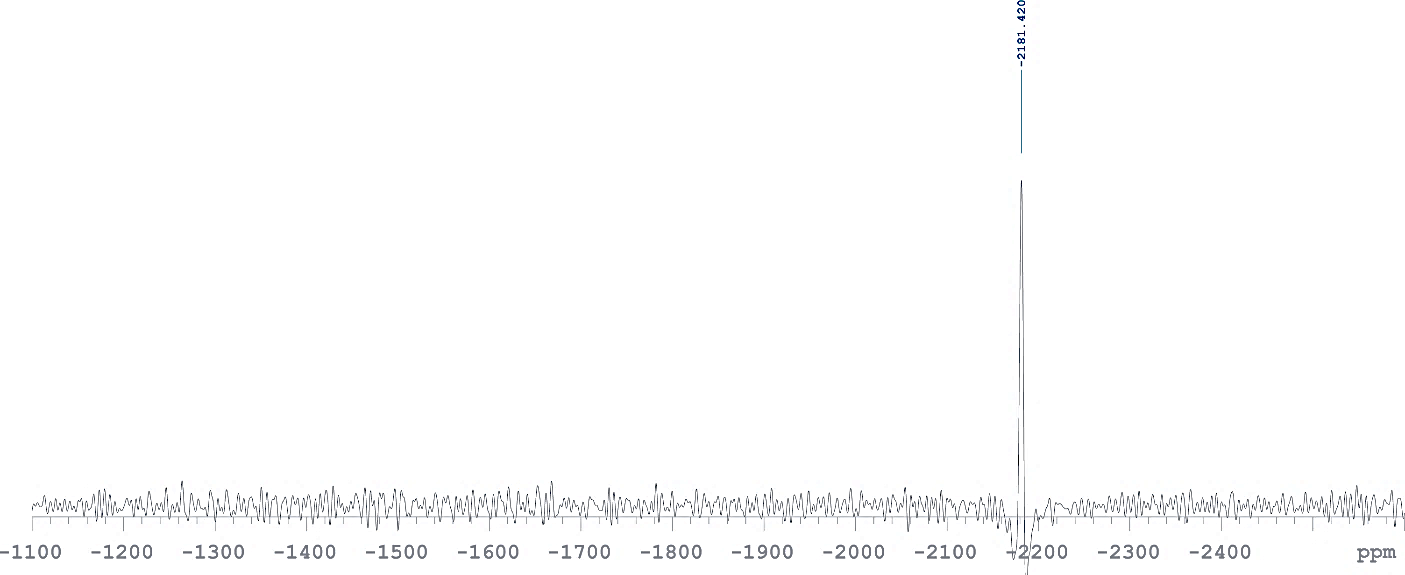


Complex **6**


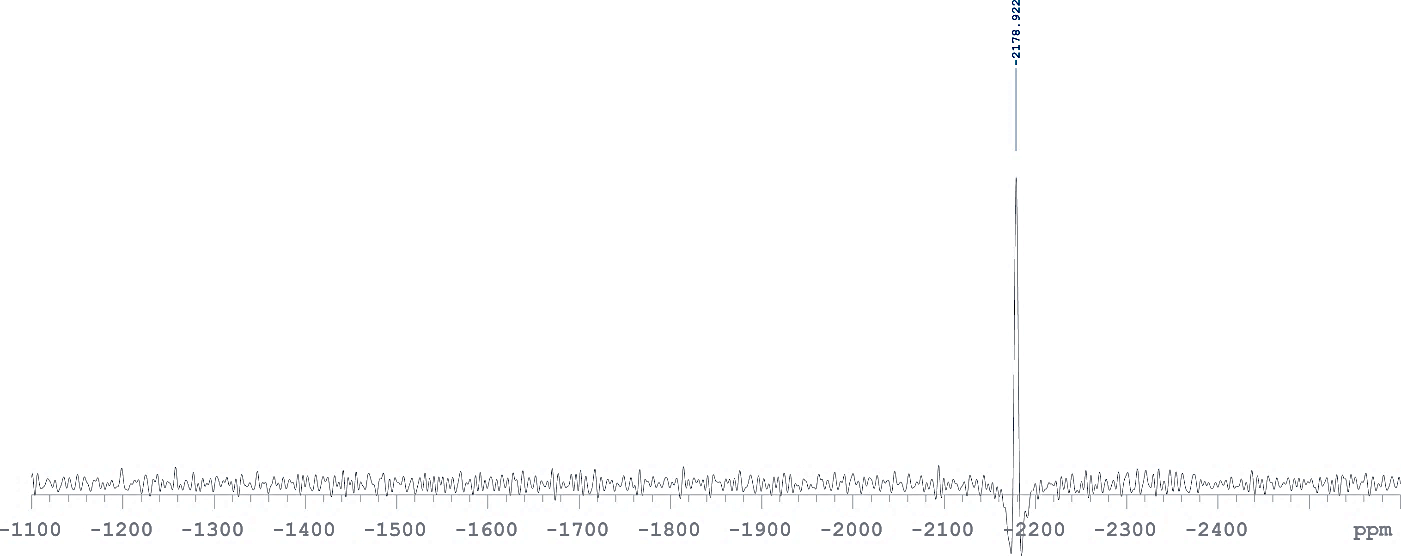


Complex **7**

**Fig. S4.2.** ^195^Pt NMR spectra of complexes **4**—**7** (D_2_O).


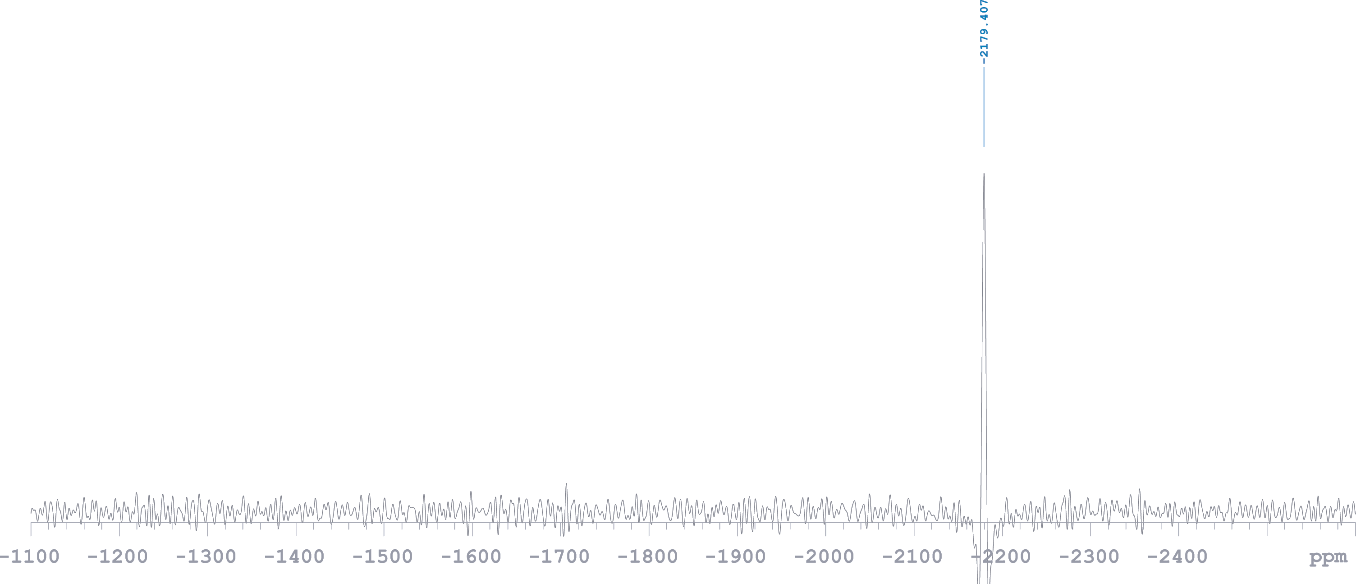


^195^Pt NMR

Complex **8**


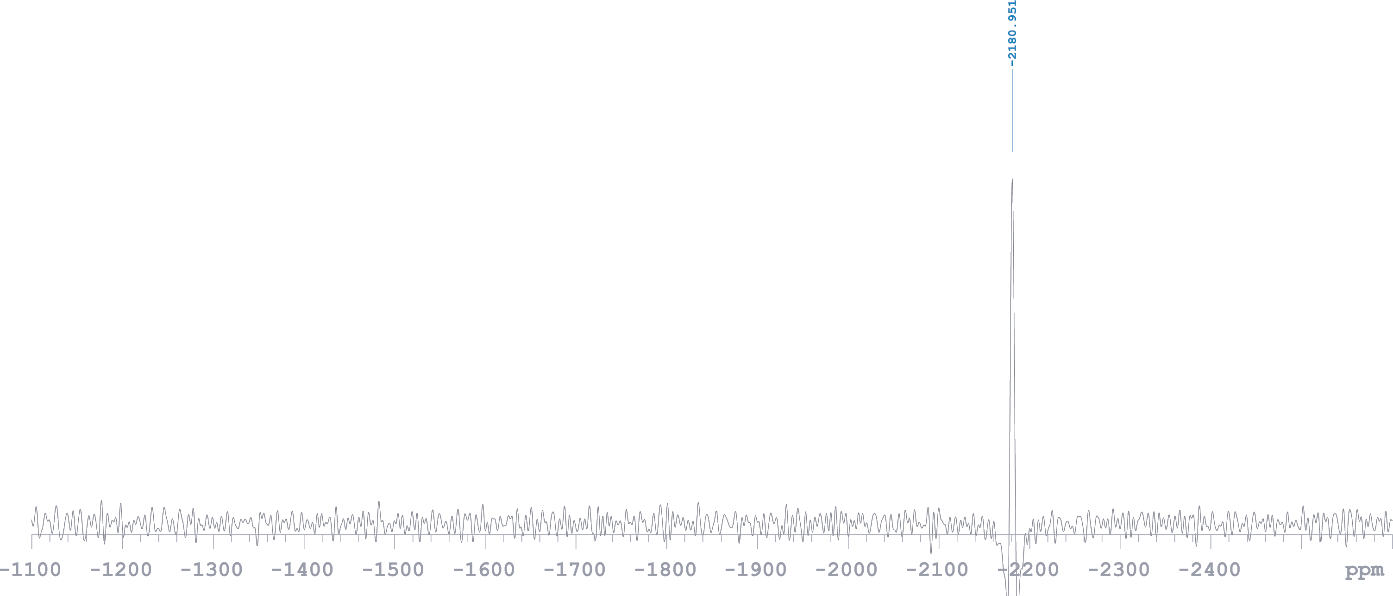


Complex **9**


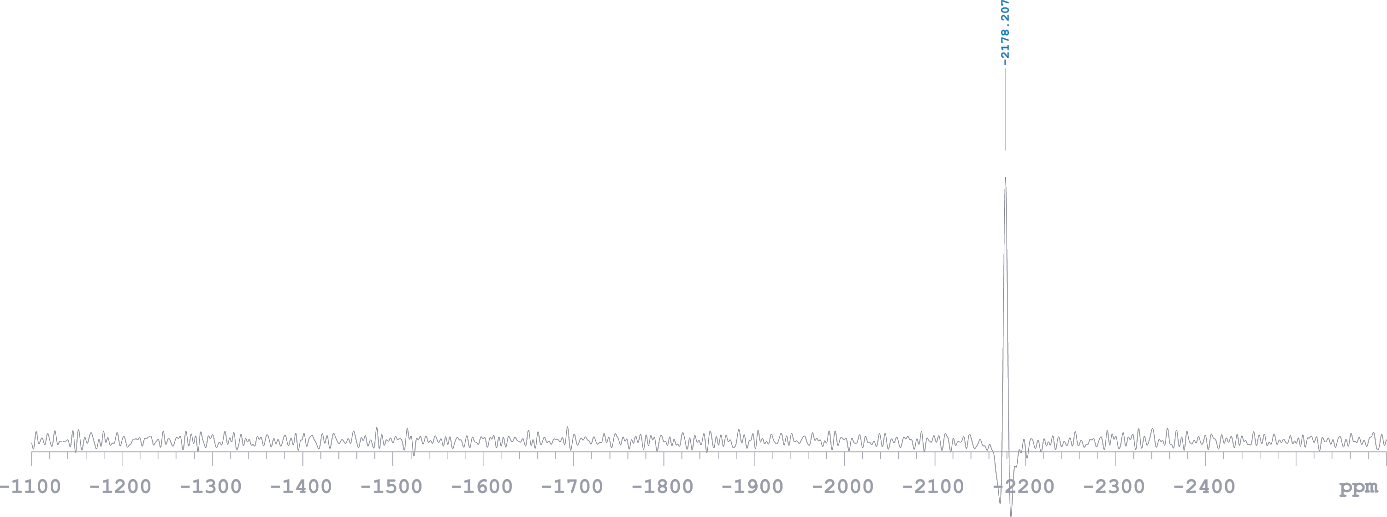


Complex **10**


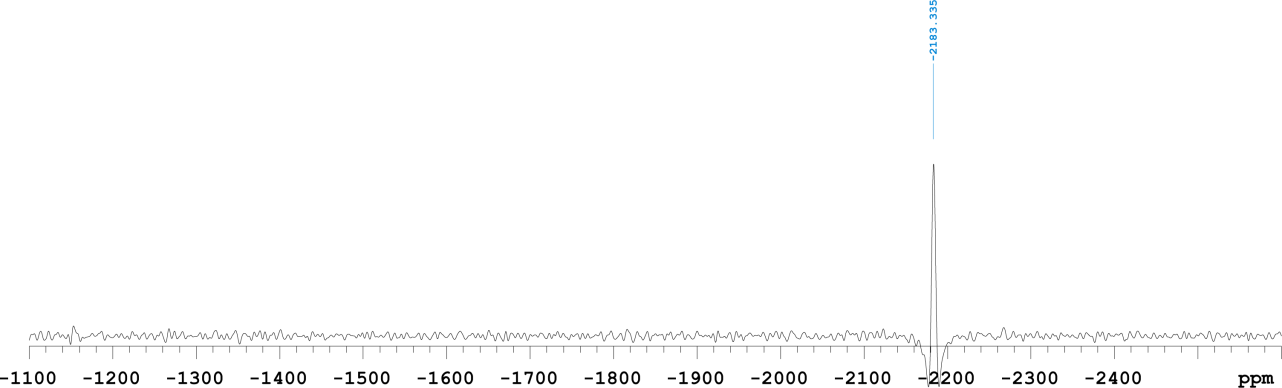


Complex **11**

**Fig. S4.3.** ^195^Pt NMR spectra of complexes **8**—**11** (D_2_O).

^195^Pt NMR


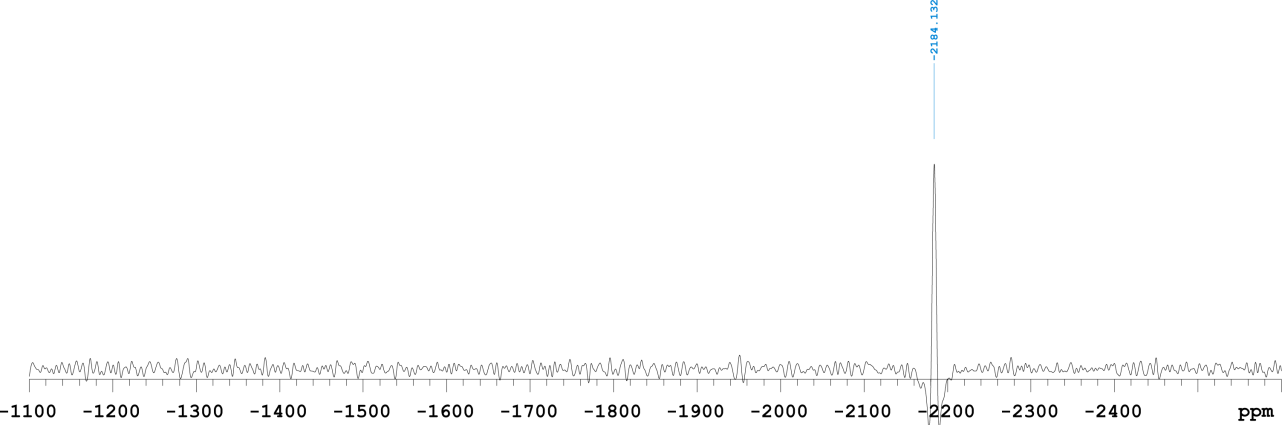


Complex **12**


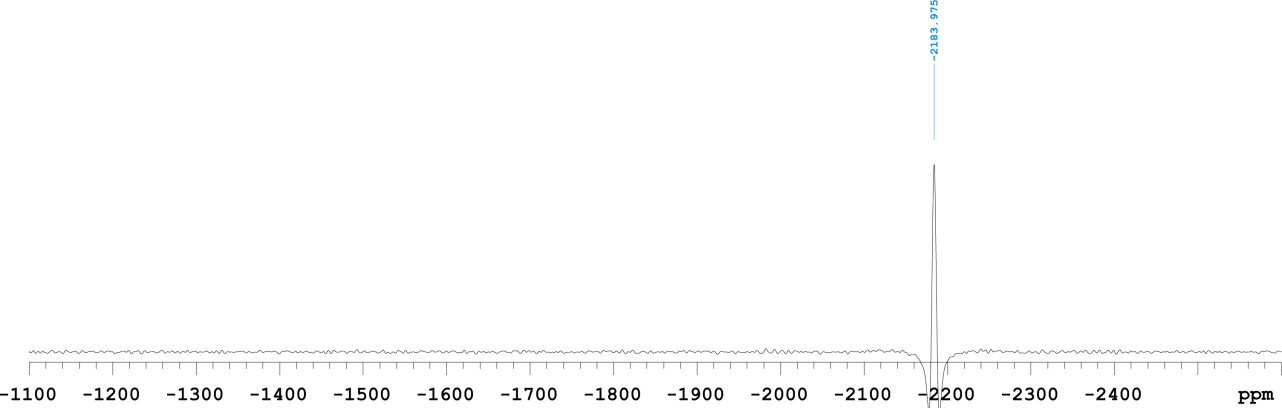


Complex **13**


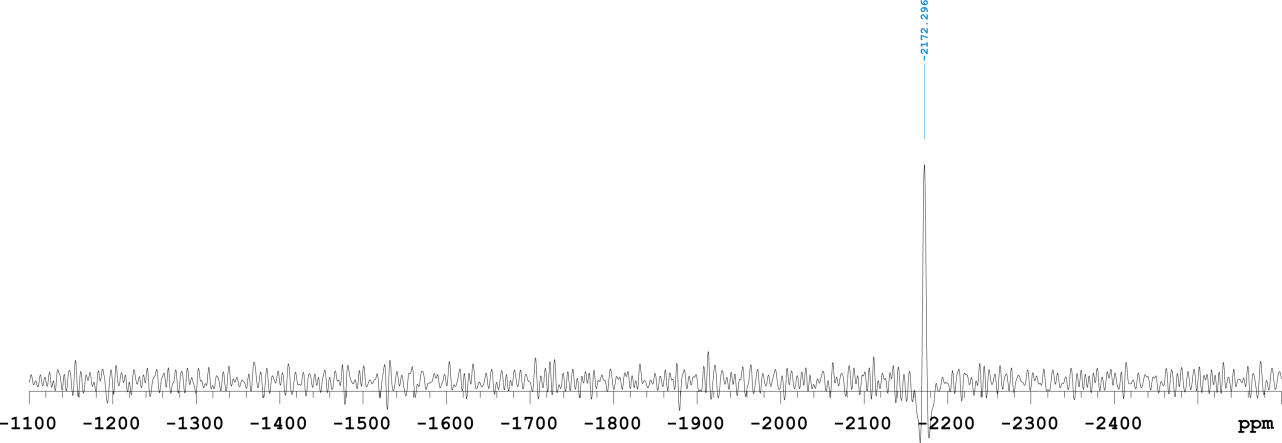


Complex **14**


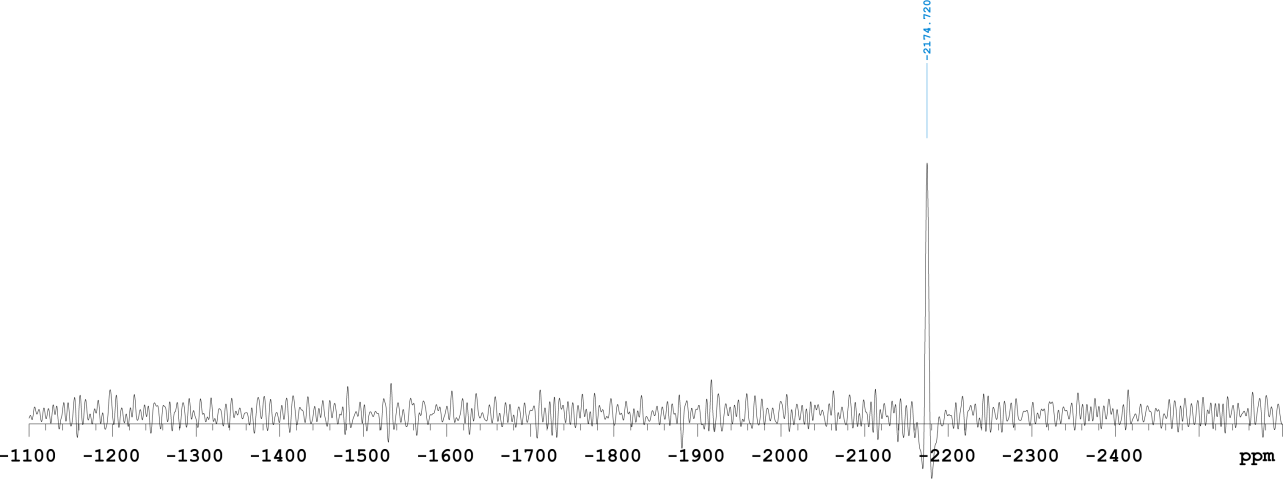


Complex **15**

**Fig. S4.4.** ^195^Pt NMR spectra of complexes **12**—**15** (D_2_O).

**
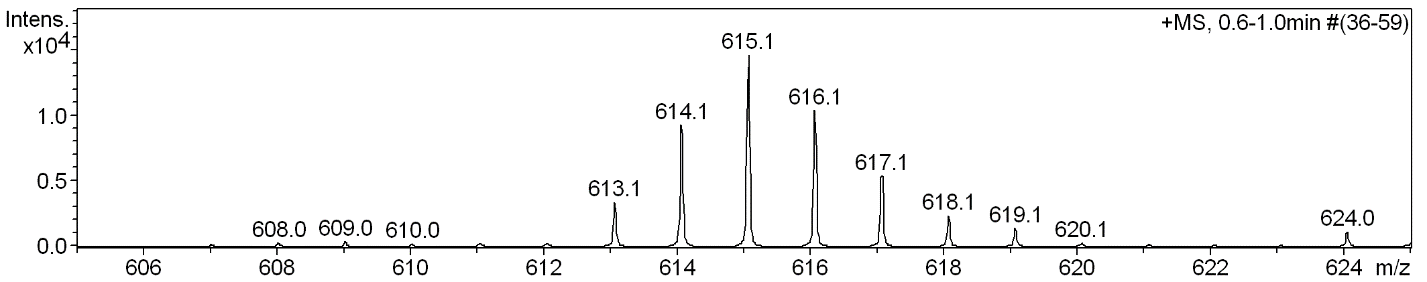
**

Complex **1**

**
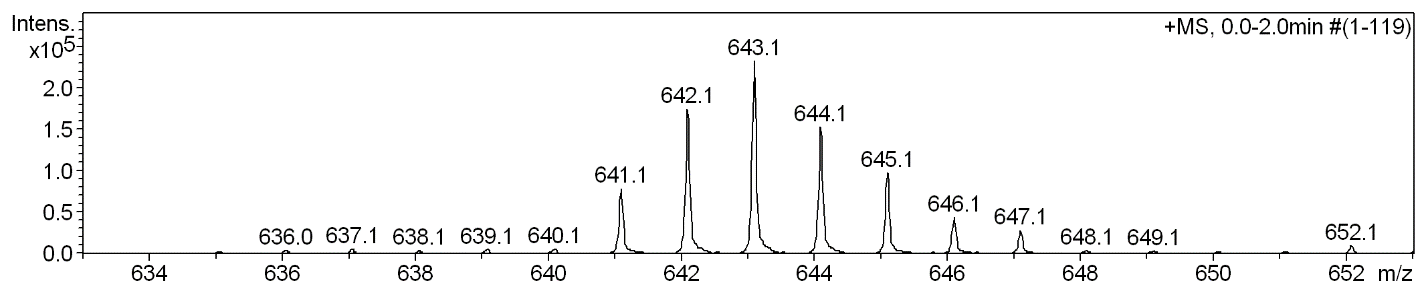
**

Complex **2**

**
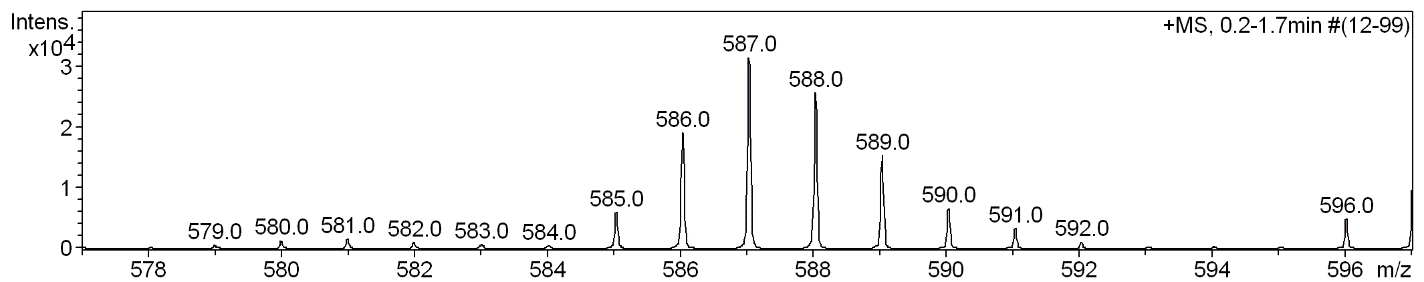
**

Complex **3**

**
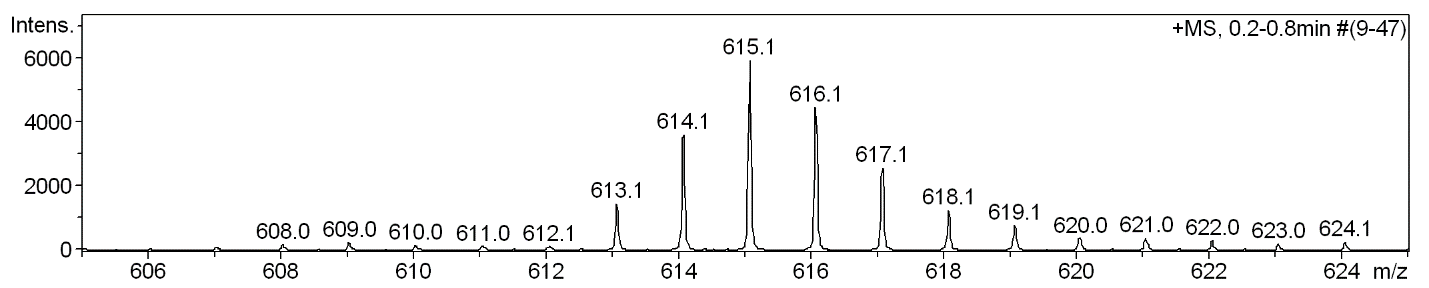
**

Complex **4**

**
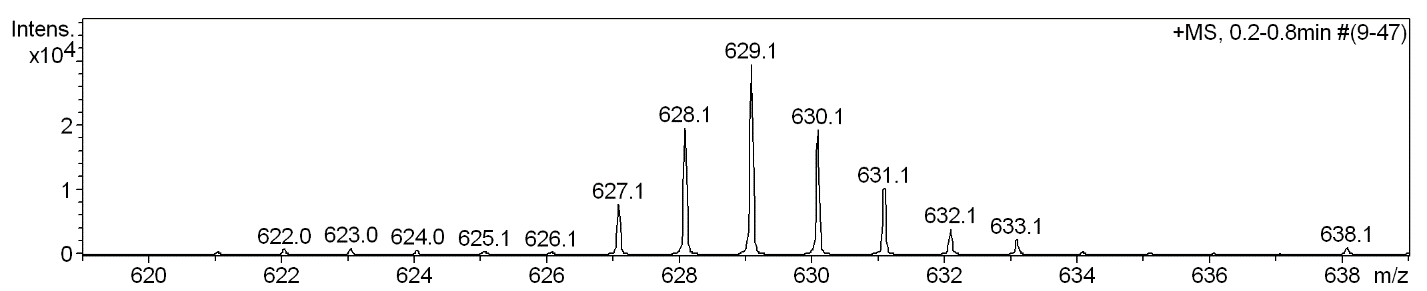
**

Complex **5**

**Fig. S5.1.** ESI-MS spectra of complexes **1**—**5**.

**
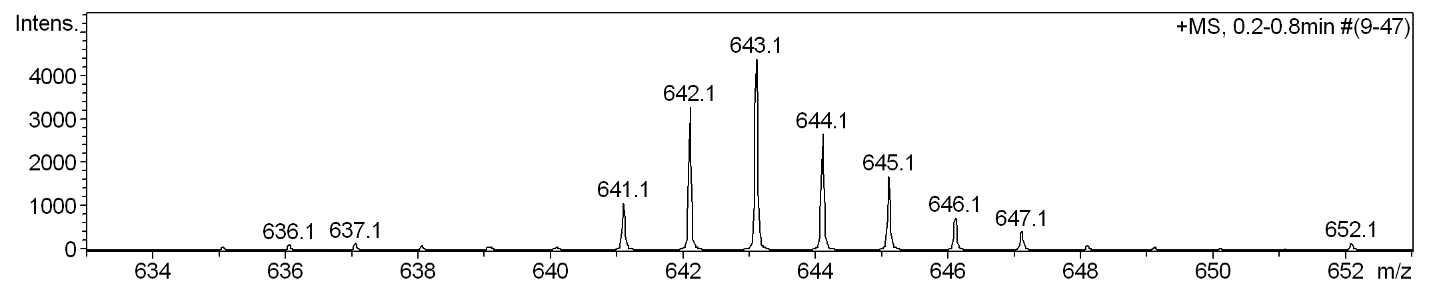
**

Complex **6**

**
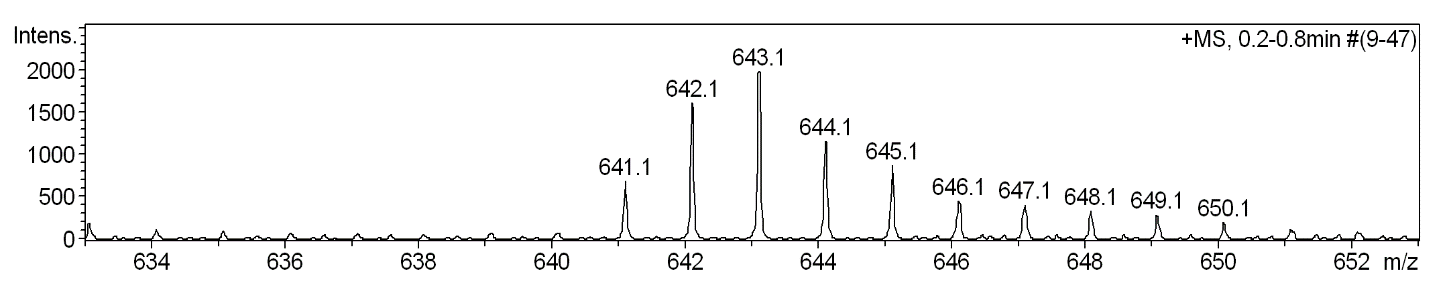
**

Complex **7**

**
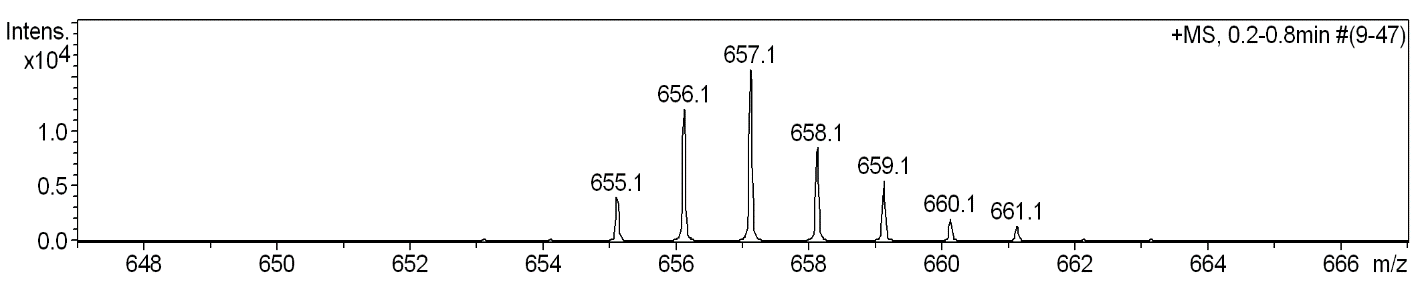
**

Complex **8**

**
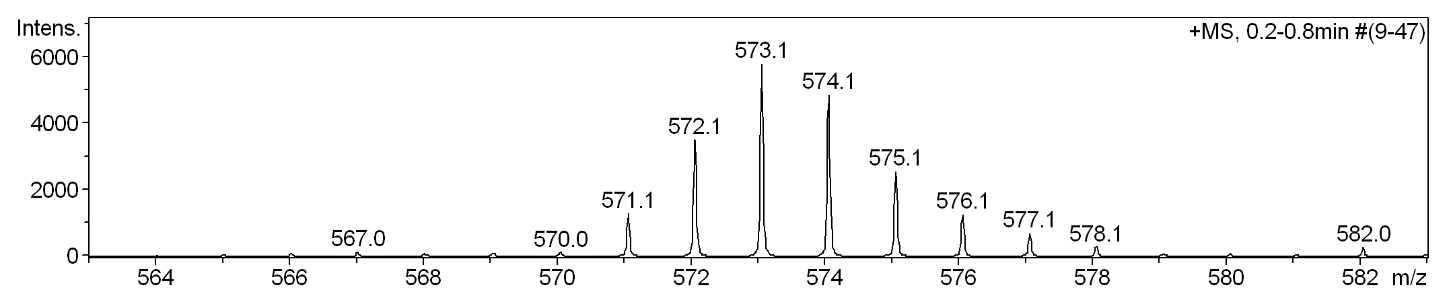
**

Complex **9**

**
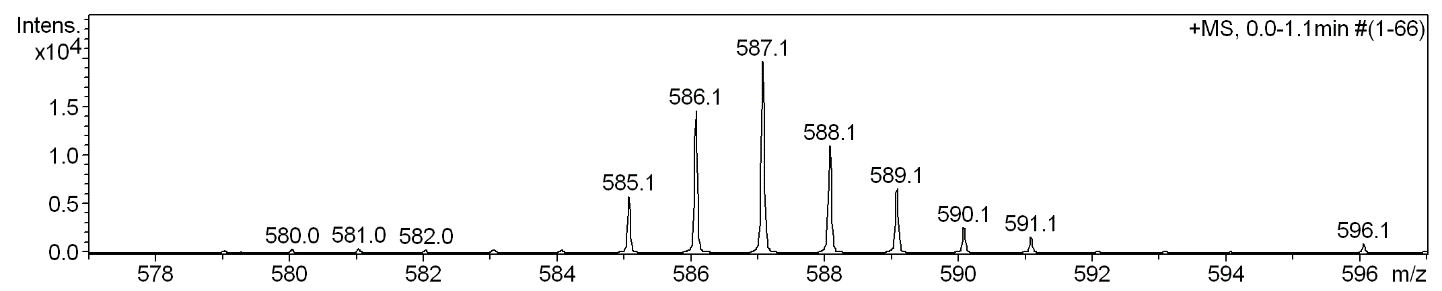
**

Complex **10**

**Fig. S5.2.** ESI-MS spectra of complexes **6**—**10**.


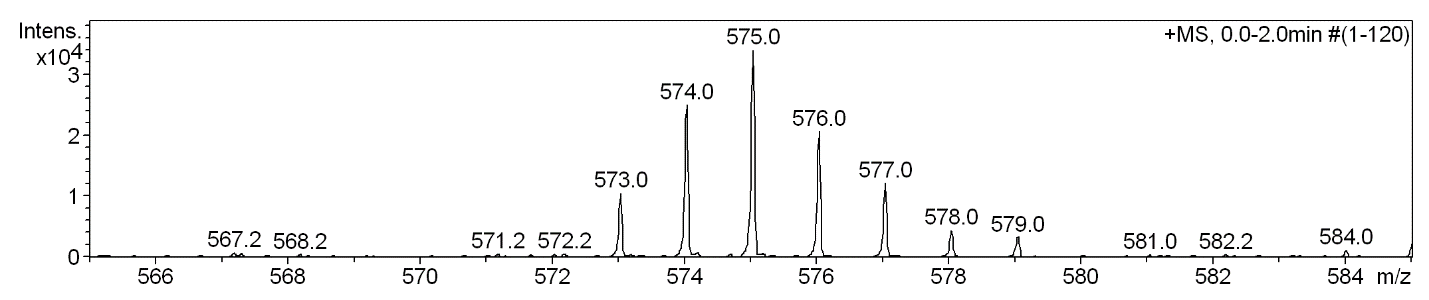


Complex **11**


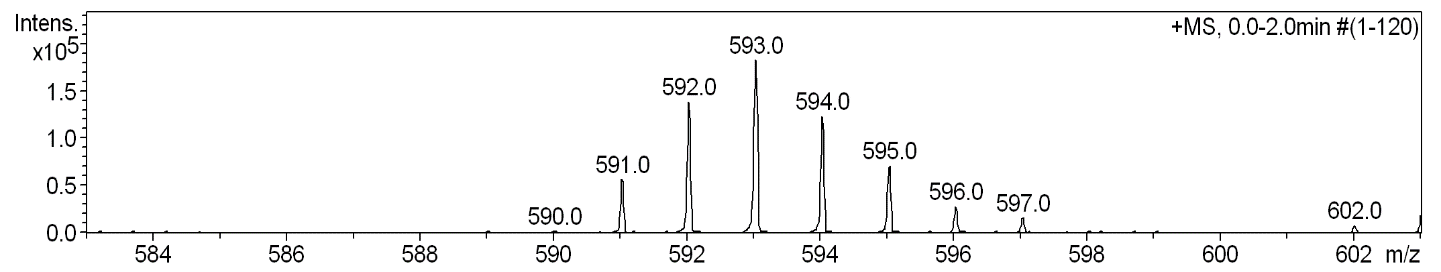


Complex **12**


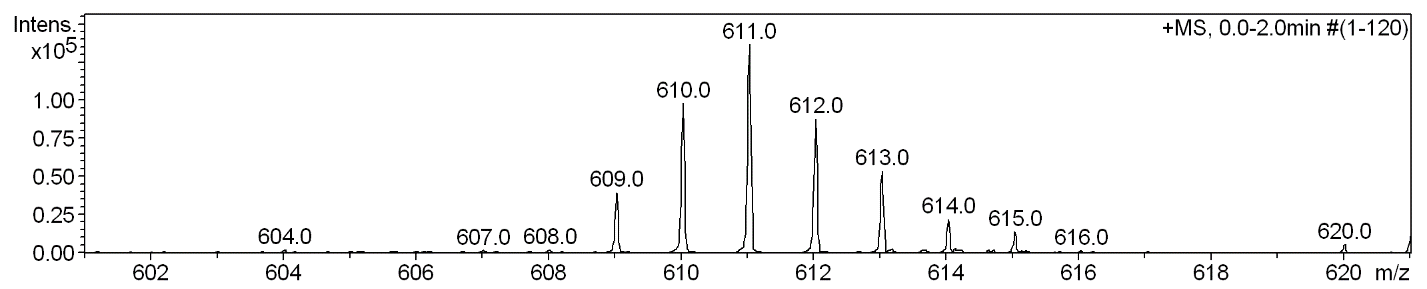


Complex **13**


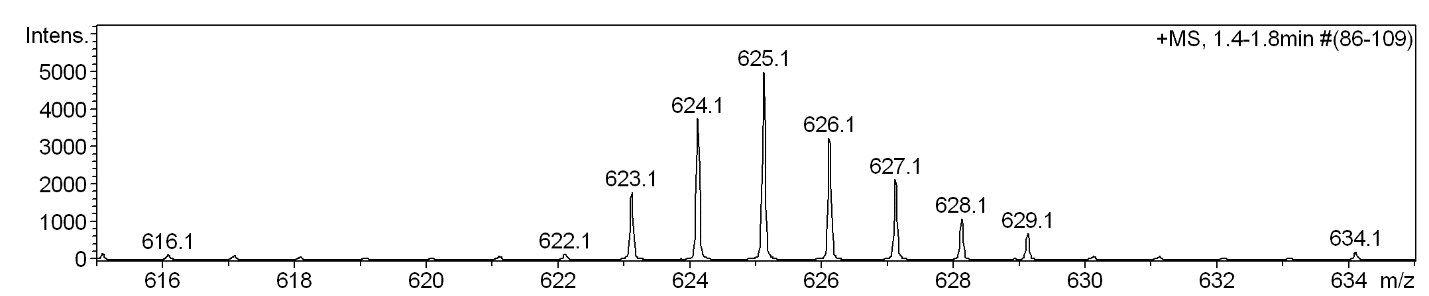


Complex **14**


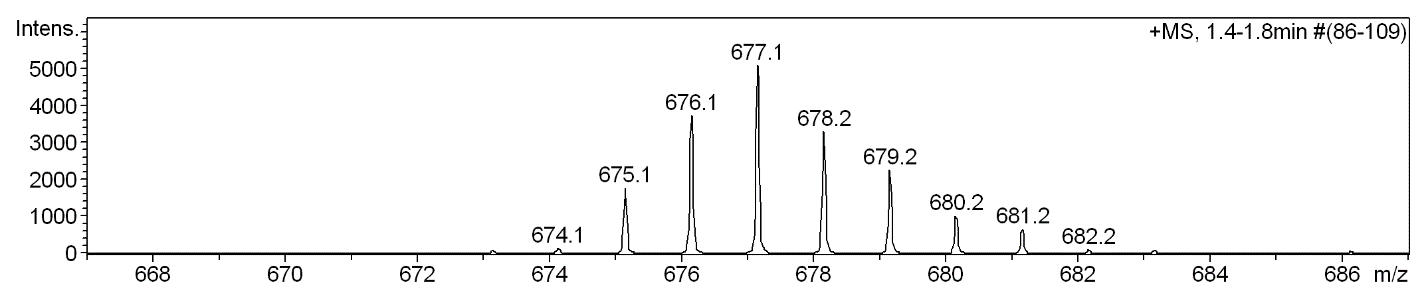


Complex **15**

**Fig. S5.3.** ESI-MS spectra of complexes **11**—**15**.
